# Optimized syntheses of Fmoc azido amino acids for the preparation of azidopeptides

**DOI:** 10.1002/psc.2968

**Published:** 2017-01-25

**Authors:** Jan Pícha, Miloš Buděšínský, Kateřina Macháčková, Michaela Collinsová, Jiří Jiráček

**Affiliations:** ^1^ Institute of Organic Chemistry and BiochemistryCzech Academy of Sciencesv.v.i., Flemingovo nám. 2166 10Prague 6Czech Republic

**Keywords:** synthesis, azido amino acid, alanine, homoalanine, ornithine, lysine, azide elimination

## Abstract

The rise of CuI‐catalyzed click chemistry has initiated an increased demand for azido and alkyne derivatives of amino acid as precursors for the synthesis of clicked peptides. However, the use of azido and alkyne amino acids in peptide chemistry is complicated by their high cost. For this reason, we investigated the possibility of the in‐house preparation of a set of five Fmoc azido amino acids: *β*‐azido l‐alanine and d‐alanine, *γ*‐azido l‐homoalanine, *δ*‐azido l‐ornithine and *ω*‐azido l‐lysine. We investigated several reaction pathways described in the literature, suggested several improvements and proposed several alternative routes for the synthesis of these compounds in high purity. Here, we demonstrate that multigram quantities of these Fmoc azido amino acids can be prepared within a week or two and at user‐friendly costs. We also incorporated these azido amino acids into several model tripeptides, and we observed the formation of a new elimination product of the azido moiety upon conditions of prolonged couplings with 2‐(1*H*‐benzotriazol‐1‐yl)‐1,1,3,3‐tetramethyluronium hexafluorophosphate/DIPEA. We hope that our detailed synthetic protocols will inspire some peptide chemists to prepare these Fmoc azido acids in their laboratories and will assist them in avoiding the too extensive costs of azidopeptide syntheses.

Experimental procedures and/or analytical data for compounds **3**–**5**, **20**, **25**, **26**, **30** and **43**–**47** are provided in the supporting information. © 2017 The Authors Journal of Peptide Science published by European Peptide Society and John Wiley & Sons Ltd.

**Abbreviations**
Bn: benzyl
Boc_2_O di‐*tert*‐butyl dicarbonate: CAS 24424‐99‐5
Fmoc‐OSu *N*‐(9‐fluorenylmethoxycarbonyloxy)succinimide: CAS 82911‐69‐1
MsCl mesyl chloride: CAS 124‐63‐0
PIDA (diacetoxyiodo)benzene: CAS 3240–34‐4
TfN_3_ triflic azide: CAS 3855‐45‐6
triflic anhydride trifluoromethanesulfonic anhydride: CAS 358‐23‐6.
Z: benzyloxycarbonyl.

## Introduction

The discovery of the CuI‐catalyzed Huisgen 1.3‐dipolar cycloaddition reaction of azides and alkynes, which leads to the formation of 1,4‐disubstituted 1,2,3‐triazoles 1, 2, has triggered a boom of these types of click reactions, which have subsequently found a plethora of applications in peptide and protein chemistry. It is because 1,2,3‐triazoles present a motif with structural and electronic characteristics similar to those of the peptide bond 3, 4. 1,2,3‐Triazoles have also found applications in cyclizations 5, 6, induction of *β*‐turns 7, 8, *β*‐hairpins 9, helical structures 10, 11 or mimics of disulfide bonds 12 in peptides. The broad applications of 1,2,3‐triazoles were reviewed in many excellent reviews (e.g. 13, 14 or 15). Moreover, azides have also found utility in a variant of Staudinger ligation for the synthesis of peptides and proteins 16. However, the syntheses of peptides with azido or alkyne moieties are often hampered by the high cost of azido or alkyne precursors, mostly Fmoc‐protected azido and alkyne amino acids, which are usually available for about €250–300 per 250 mg. This can make the preparation of larger series of azido/alkyne peptides very expensive, as we have recently experienced 17. For this reason, we decided to investigate the accessibility of the in‐house preparation of a series of five Fmoc‐protected azido amino acids; (*S*)‐2‐amino‐3‐azidopropanoic and (*R*)‐2‐amino‐3‐azidopropanoic acids (*β*‐azido l‐alanine and d‐alanine), (*S*)‐2‐amino‐4‐azidobutanoic acid (*γ*‐azido l‐homoalanine), (*S*)‐2‐amino‐5‐azidopentanoic acid (*δ*‐azido l‐ornithine) and (*S*)‐2‐amino‐6‐azidohexanoic acid (*ω*‐azido l‐lysine).

Serine is a convenient precursor for the synthesis of the azido derivative of alanine. Several different methods were previously developed for introducing the azido moiety to a serine derivative: (i) ring opening of cyclic *N*‐(phenyl fluoride) serine sulfamidate 18, 19, 20, (ii) opening of (*S*)‐3‐amino‐2‐oxetanone 21, (iii) mild bromination of the hydroxyl group followed by azidation 22, 23, 24, 25, (iv) Mitsunobu reaction treatment with triphenylphosphine (PPh_3_), hydrazoic acid and azodicarboxylate 26, 27, 28, 29, 30, 31, 32, 33, 34, 35, and (v) activation of the hydroxyl group by mesyl chloride (MsCl) 35, 36, 37, 38 or tosyl chloride 39, followed by substitution with sodium azide. In addition, the synthesis of azidoalanine starting from *N*‐protected asparagine represents a different but straightforward approach. The first step is Hoffman degradation by treatment with *I*‐bis(trifluoroacetate) (CAS 2712‐78‐9) 40, 41, 42, 43, 44 or (diacetoxyiodo)benzene (PIDA) 45, followed by a diazotransfer reaction 41, 46, 47, 48, 49, 50, 51.

In general, there are two main approaches for the synthesis of derivatives of Fmoc‐l‐azidohomoalanine. Mostly, the protected Fmoc‐l‐glutamine is converted under Hofmann rearrangement conditions 41, 52, 53, 54 to 2‐Fmoc‐4‐aminobutanoic acid, followed by the azido transfer reaction 12, 41, 46. The other approach involves protected l‐aspartic acid, which is partially reduced via a mixed anhydride. The resulting alcohol is mesylated, and the corresponding mesyl derivative is replaced by the azido function 55.

The strategy of orthogonal functional group protection 56, 57, 58, 59 of l‐ornithine and l‐lysine is usually used for the synthesis of their azido derivatives.

Here, we compared several of these reaction pathways and also investigated a few new routes for the preparation of the above‐listed Fmoc‐protected azido amino acids in a multigram scale, including their incorporation into short model peptides. The advantages and drawbacks of the approaches are discussed.

## Results and Discussion

### Synthesis of β‐Azido l‐Alanine and d‐Alanine

**Scheme 1:**
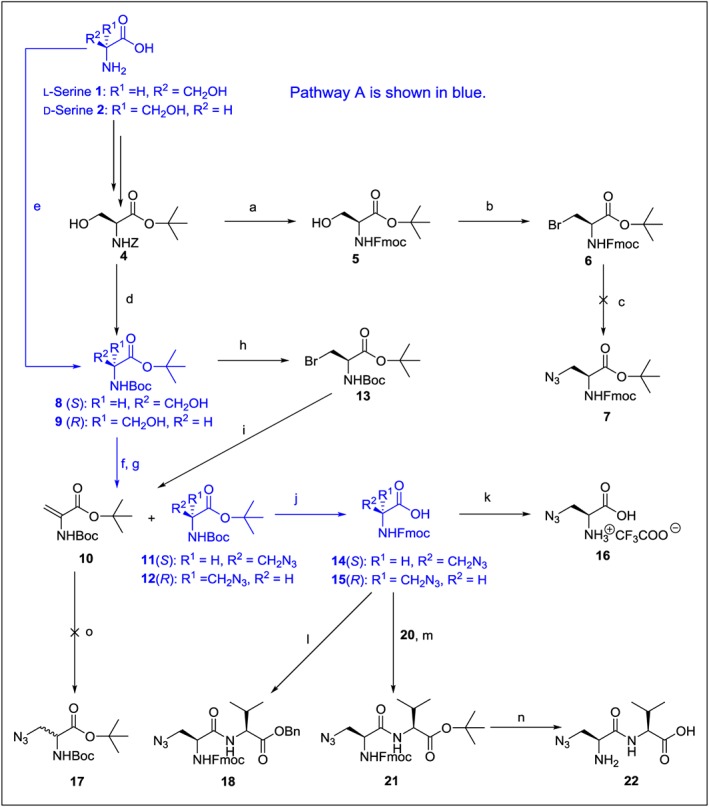
Reagents, conditions and yields: (a) 10% Pd/C, H_2_, methanol, RT, overnight then Fmoc‐OSu, NaHCO_3_, water and dioxane, 0 °C for 1 h, then RT overnight (74% over two steps); (b) CBr_4_, PPh_3_, DCM 0 °C for 1 h, then RT overnight (84%); (c) Trimethylsilyl azide, hexamethylphosphoramide, 70 °C overnight; (d) 10% Pd/C, H_2_, methanol, RT overnight, then Boc_2_O, NaHCO_3_, water and dioxane, 0 °C for 1 h, then RT overnight (86% over two steps); (e) Boc_2_O, NaHCO_3_, water and dioxane, 0 °C for 1 h, followed at RT overnight, then C_4_H_9_Br, K_2_CO_3_, benzyl triethylammonium chloride, *N*,*N*‐dimethyl acetamide, 60 °C overnight (72% over two steps for **8**, 67% over two steps for **9**); (f) MsCl, TEA, DCM, 0 °C for 0.5 h, then NaN_3_, DMSO, 70 °C overnight (16% for **10**, 47% for **11** over two steps, 21% for **10**, 50% for **12** over two steps); (g) MsCl, pyridine, DCM, 0 °C for 0.5 h, then NaN_3_ DMSO, 70 °C overnight (26% for **10**, 46% for **11** over two steps); (h) CBr_4_, PPh_3_, DCM, 0 °C for 1 h, then RT overnight (68%); (i) NaN_3_, DMSO, 70 °C overnight (40% for **10**, 41% for **11** over two steps); (j) TFA, DCM, water, RT overnight, then NaHCO_3_ and Fmoc‐OSu, dioxane and water, 0 °C for 1 h, then RT overnight (86% for **14** over two steps, 86% for **15** over two steps); (k) 2‐chlorotrityl chloride resin, DIPEA, DMF, 2 h, then piperidine, DMF (75%); (l) l‐Val‐OBn·TsOH, DIPEA, PyBroP, DMF RT overnight (74%); (m) **20**, DIPEA, PyBroP, DMF, RT overnight (70%); (n) TFA, DCM, 2 h, 2‐chlorotrityl chloride resin, DIPEA, DMF, 2 h, then piperidine, DMF (67%); (o) NaN_3_, DMSO, 70 °C overnight.

Firstly, l‐serine **1** was chemoselectively protected by the benzyloxycarbonyl (Z) group, and Z‐Ser **3** was then esterified with *tert*‐butyl bromide in the presence of a benzyltriethylammonium chloride phase catalyst and excess of potassium carbonate 60, 61 (Scheme 1). These two transformations led to the intermediate **4**. The removal of the Z group by a catalytic hydrogenation and a subsequent acylation with *N*‐(9‐fluorenylmethoxycarbonyloxy)succinimide (Fmoc‐OSu) yielded Fmoc‐protected compound **5**, which, after the Abell reaction conditions, gave compound **6**. It is well known that the direct azidation of bromo compounds in DMF (CAS 68‐12‐2) is unfeasible because of the high basicity of NaN_3_. Therefore, for the introduction of N_3_ moiety under mild conditions, we decided to apply trimethylsilyl azide (CAS 4648‐54‐8) in hexamethylphosphoramide (CAS 680‐31‐9) or DMF 62, 63. However, we surprisingly only isolated from the reaction mixture starting compound **6** instead of **7**. The failure of this synthetic pathway indicated that the sequence of steps must be changed; the azido moiety should be introduced first, followed by the deprotection of the amino group and then by derivatization with Fmoc. Thus, the Z group was again subjected to catalytic hydrogenolysis, and then the free amine group was protected by Boc to afford derivative **8**. We also found that the key intermediate **8** can be achieved by a shortcut (Scheme 1, pathway A) directly from l‐serine **1**. Thus, the Boc‐l‐Ser‐O*t*Bu **8** was prepared in a good yield (72%) by the reaction of l‐serine with *tert*‐BuBr in a similar manner as compound **4**. Thereafter, activation of the hydroxyl group was performed with MsCl under basic conditions (TEA, CAS 121‐44‐8), ensuring the stability of acid‐sensitive groups. The reaction mixture was tested with TLC, and except for the required mesyl intermediate **11**, the dehydroalanine **10** was also observed as the elimination product 31, 64. We also tested pyridine as a weaker base instead of TEA, but the full conversion of **8** to mesyl intermediate **11** was achieved only after 24 h, but again accompanied by a significant formation of **10**. To prevent this unwanted process, compound **8** was transformed to bromide derivative **13** and subsequently reacted with sodium azide in DMSO (CAS 67‐68‐5). In this case, **11** was obtained in a similar yield, but with a substantially higher amount of the elimination product **10**. Probably, a more easily leaving Br^−^ group better promotes the elimination process. Hence, we can conclude that both mesylation and azidation reactions contribute to the formation of dehydroalanine **10**. Finally, with the use of standard procedures, both acid‐labile protecting groups of **11** were removed with TFA (CAS 76‐05‐1) and the amino group was acylated with Fmoc‐Osu to furnish the required product **14**.

Next, starting from d‐serine **2** and through intermediates **9** and **12**, we employed the synthetic pathway A and the following reactions for the preparation of Fmoc‐*β*‐azido‐d‐Ala **15**.

Cumulative yields of the six‐step synthetic pathway A (i.e., (i) acylation with Boc, (ii) alkylation with *tert*‐But, (iii) activation by Ms, (iv) addition of azide, (v) elimination of acid‐labile groups and (vi) acylation with Fmoc) were satisfactory: 29% for **14** and 28% for **15**, both in multigram scales.

Johansson and Pedersen 65 and others 35, 66 claimed that dehydroalanine **10** is a perfect Michael acceptor, which undergoes racemization. To verify this statement, we carried out a simple experiment; the isolated dehydroalanine **10** was heated in DMSO with an excess of NaN_3_ at 70 °C overnight. However, no traces of the expected product **17** were found.

Next, we verified the optical purity of the compound **14** by NMR spectroscopy. For this, the dipeptide **18** was prepared by the reaction of **14** with l‐Val‐OBn·TsOH. The incorporation of valine added a new stereo center to the molecule. However, the presence of the sterically bulky Fmoc group resulted in the observation of geometrical isomers of the carbamate in a ratio of 60 : 40. Therefore, we had to prepare a fully deprotected molecule. Compound **14** was thus coupled with ester **20**, which was prepared by esterification of l‐valine **19** using *tert*‐butyl acetate 67 as a source of (CH_3_)_3_C^+^ cation. Acidic hydrolysis gave free acid, which was attached to 2‐chlorotrityl chloride resin. This allowed the removal of the Fmoc protecting group by conveniently washing off poorly separable dibenzofulvene. A usual work‐up and separation furnished dipeptide **22**, which was manifested by only one set of signals in both ^1^H and ^13^C NMR spectra. The same protocol with the chlorotrityl resin was used for the preparation of free amino acid **16**. The optical purity of water‐soluble acid **16** was checked by the method of Inamoto *et al*. 68. No splitting of signals on *α*‐carbons and *α*‐protons was observed after the addition of sodium [(*S*)‐1,2‐diaminopropane‐*N*,*N*,*N*′,*N*′‐tetraacetato]‐samarate(III) 69 to a pH‐adjusted solution of **16** in a 2 : 1 molar ratio. In conclusion, NMR analyses unequivocally proved the high optical purity of compound **14**. We have not performed the same procedure with the optical isomer **15**, which was prepared by the same reactions, but from the pure d‐serine. However, the same high optical purity can be expected.

**Scheme 2:**
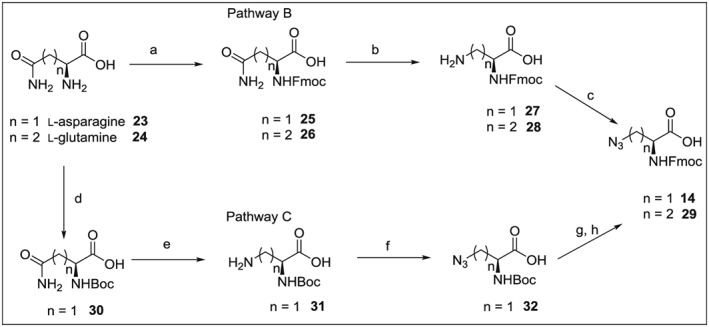
Reagents, conditions and yields: (a) Na_2_CO_3_, Fmoc‐OSu, dioxane and water, 0 °C for 1 h, then RT overnight (87% for **25**, 93% for **26**); (b) PhI(OAc)_2_, CH_3_CN, ethyl acetate and water at RT overnight (75% for **27**, 56% for **28**); (c) TfN_3_, NaHCO_3_, CuSO_4_·5H_2_O, water and methanol at RT overnight (80% for **14**, 92% for **29**); (d) Na_2_CO_3_, Boc_2_O, dioxane and water 0 °C 1 h then RT overnight (73%); (e) PhI(OAc)_2_, CH_3_CN, ethyl acetate and water at RT overnight (75%); (f) TfN_3_, TEA, CuSO_4_·5H_2_O, water and methanol at RT overnight; (g) TFA, DCM, 2 h at RT; (h) NaHCO_3_, Fmoc‐OSu, dioxane and water, 0 °C for 1 h, then RT overnight (37% over three steps).

We also investigated two alternative reaction routes (Scheme 2) for the synthesis of Fmoc‐*β*‐azido‐l‐Ala **14** as outlined in Scheme 2. Firstly, l‐asparagine **23** was protected by the Fmoc group; then carboxamide functionality of the resulting intermediate **25** was eliminated under Hoffmann rearrangement conditions. The last step of this reaction pathway B was a diazotransfer reaction, which allowed the conversion of the amino group to the corresponding azido acid **14**. In parallel, we demonstrated that **14** can be obtained with a similar synthetic strategy, but using Boc protection and starting from l‐asparagine **23** over intermediates **31** and **32** (reaction pathway C).

Compound **14**, which was prepared using three different synthetic pathways (A, B or C), provided the same mass and NMR spectra and other physicochemical characteristics. Therefore, the number of synthetic steps, cumulative yields and costs are decisive for the choice of the optimal strategy. Pathway A includes six steps and gave 29% yield, pathway B was performed in three steps and with 52% yield and pathway C required five steps and gave 20% yield. Clearly, from this aspect, the preferred synthetic route is pathway B and the less convenient is pathway C. We also calculated the approximate costs of synthetic pathways A and B for the preparation of **14**, using precursor and solvents purchased at standard prices from Fluka. Synthetic pathway A yielded 1 g of **14** for about €37 and pathway **B** for about €43. Pathway A can be completed within a week; pathway B is faster. Taking everything together, the method of choice for the preparation of **14** is pathway B, despite the fact that its diazotransfer reaction requires the use of an excess of rather costly trifluoromethanesulfonic anhydride (triflic anhydride).

### Synthesis of *β*‐Azido l‐Homoalanine

Next, for the preparation of l‐homoazidoalanine, we chose the straightforward pathway B (Scheme 2), starting from l‐glutamine over intermediates **26** and **28**. The product **29** was obtained in an excellent yield of 92%.

### Synthesis of *δ*‐Azido l‐Ornithine and *ω*‐Azido l‐Lysine

**Scheme 3:**
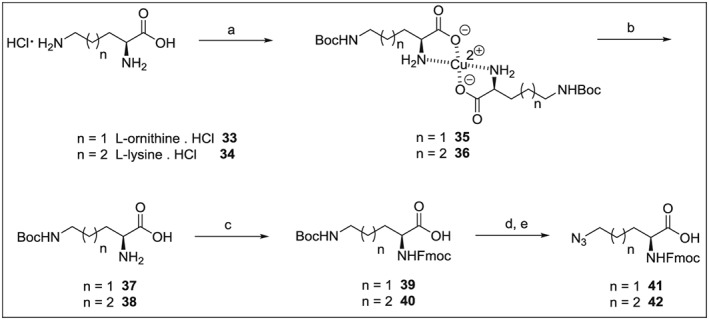
Reagents, conditions and yields: (a) CuAc_2_·H_2_O, water, then Boc_2_O in acetone overnight (90% for **35**, 92% for **36**); (b) 8‐hydroxyquinoline, water, 4 h (90% for **37**, 88% for **38**); (c) Fmoc‐OSu, NaHCO_3_, water and dioxane, 0 °C for 0.5 h, then RT overnight (93% for **39**, 95% for **40**); (d) TFA, DCM, 2 h at RT (e) TfN_3_, NaHCO_3_, CuSO_4_·5H_2_O, water and methanol at RT overnight (92% for **41** over two steps, 89% for **42** over two steps).

For the synthesis of azido acids **41** and **42**, derivatives of l‐ornithine and l‐lysine, we applied the strategy of orthogonal functional group protection 56, 57, 58, 59 (Scheme 3). Synthesis started from commercially available l‐ornithine·HCl **33** or l‐lysine·HCl **34**, and the reaction with copper acetate monohydrate under basic conditions afforded [Orn(Boc)]_2_Cu **35** or [Lys(Boc)]_2_Cu **36**, respectively, which were isolated by a perfect filtering off of their insoluble copper complexes. Metal was quantitatively removed using 8‐quinolinol to furnish selectively Boc‐protected intermediates **37** and **38** in forms of zwitterions. The alpha amino group was acylated with Fmoc‐OSu, and the resulting diamino acids **39** and **40** were treated with TFA to liberate *δ*‐free amine and *ω*‐free amine, respectively. The final step is represented by a diazotransfer reaction, which leads to the required Fmoc‐azido‐l‐norvaline **41** or Fmoc‐azido‐l‐lysine **42**.

### Synthesis of Model Azido Tripeptides

**Scheme 4:**
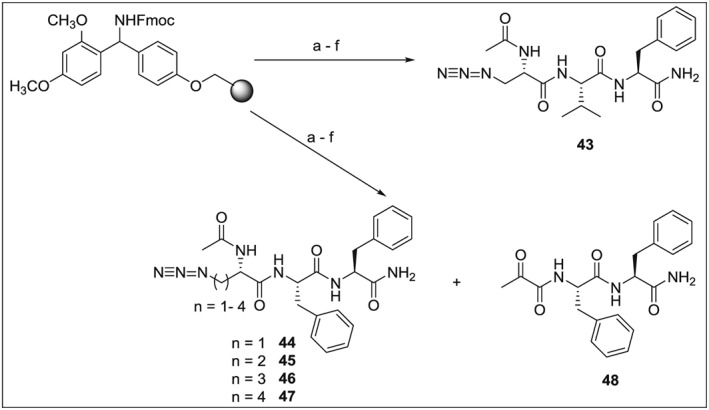
Reagents, conditions and yields: (a) 20% piperidine/DMF; (b) (i) Fmoc‐l‐Phe, HBTU, DIPEA, DMF, 2 × 2 h at RT (ii) 20% piperidine/DMF; (c) (i) Fmoc‐l‐Phe or Fmoc‐l‐Val, HBTU, DIPEA, DMF, 2 × 2 h at RT (ii) 20% piperidine/DMF; (d) **14**, **29**, **41** or **42**, HBTU, DIPEA, DMF, 2 × 2 h at RT (ii) 20% piperidine/DMF; (e) Ac_2_O, DIPEA, DMF, 15 min at RT; (f) 95% TFA/water, 1 h at RT. Yields (after HPLC purification) 69% for **43**, 76% for **44**, 80% for **45**, 77% for **46** and 71% for **47**. In the case of long couplings (5 and 18 h) with **14**, the yield for **44** was only 17% accompanied by the presence of **48** in 32% yield.

**Figure 1:**
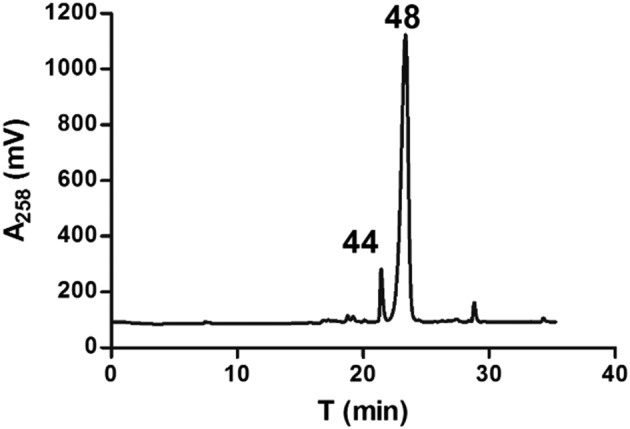
HPLC trace of the purification of the crude tripeptide **44**. The major isolated products (**44** and **48**) are labeled with compound numbers.

Finally, we synthesized a series of model tripeptides **43**–**47**, using the standard manual Fmoc solid‐phase synthesis protocol 70 (Scheme 4). When the methodology was precisely followed, all required peptides were obtained in good yields and with a high chemical purity. However, surprisingly, during the synthesis of **44** and only in the case of prolonged condensations (5 and 18 h) of Fmoc‐azidoalanine **14** with the resin‐bound Phe‐Phe‐NH_2_, we observed the massive appearance of a new compound representing a major product of the synthesis (Figure 1). This product was isolated, and its chemical structure assigned using spectral methods and attributed to the compound **48**. It appears that only alpha‐keto functionality is present in **48** instead of methylazido and acetylamino moieties. We have not found any information in the available literature about this type of elimination of azido species. Some studies (e.g., 71) reported on obtaining carbonyl compounds from *α*,*β*‐disubstituded compounds. However, here, we can only speculate that the shorter side chain of **14** (i.e., the proximity of the azido moiety and the primary *α*‐amino group) and/or possibly also the activating agent 2‐(1*H*‐benzotriazol‐1‐yl)‐1,1,3,3‐tetramethyluronium hexafluorophosphate (HBTU) in the presence of DIPEA (7087‐68‐5) and/or longer couplings or treatment with 95% TFA/5% water may play a role in this side reaction. Interestingly, in addition, it seems that this elimination is also sequence specific, as it occurred only in the case of the coupling of **14** to Phe‐Phe dipeptide and not to other sequences. Hence, at this stage, we rather do not suggest any plausible reaction mechanism for this process. The susceptibility of **14** to the elimination of its azido moiety during the synthesis of a simple tripeptide suggests that the use of azido amino acids in the peptide synthesis is not without risks and that some precautions should be taken (e.g., shorter reaction times and alternative reagents)

## Conclusions

In conclusion, we investigated several synthetic protocols for the preparation of l‐Fmoc‐*β*‐azidoalanine and d‐Fmoc‐*β*‐azidoalanine (**14** and **15**, respectively). We found that pathway B starting from asparagine is the most straightforward one and it can also be used for the preparation of *γ*‐azido l‐homoalanine when starting from glutamine. NMR analysis confirmed the high optical purity of **14** prepared with these protocols. We also synthesized l‐Fmoc‐*γ*‐azidohomoalanine **29**, l‐Fmoc‐*δ*‐azidoornitine **41** and l‐Fmoc‐*ε*‐azidolysine **42**. Several synthetic steps previously described in literature were improved and optimized, and several new reactions investigated. All synthetic procedures were described in detail, and the complete physicochemical characterization of all intermediates and final compounds was provided. We found that multigram quantities of these Fmoc‐protected azido amino acids can be prepared within a week, at average costs of about €40 per gram of final compounds (excluding work and energies). This makes them incomparably cheaper than standard commercial counterparts. We also observed a new type of elimination which occurred during prolonged couplings upon the solid‐phase synthesis of Ac‐*β*‐azido‐Ala‐Phe‐Phe‐NH_2_.

## Experimental Part

Reagents and solvents (Sigma‐Aldrich‐Fluka, St. Louis, MO, USA) used in this study were of analytical grade. TLC analyses were performed on silica‐gel‐coated aluminum plates (Fluka). The compounds were visualized by exposure to UV light at 254 nm and by ninhydrin spraying (dark blue color) of Boc‐protected amines or amines. Flash chromatography purifications were carried out on silica gel (40–63 μm, Fluka). Preparative RP‐HPLC chromatography was carried out on a C18 Luna column (Phenomenex, Torrance, CA, USA, 250 × 21.2 mm, 10 μm) at a flow rate 9 ml/min (solvent A: 0.1% TFA; solvent B: 80% CH_3_CN, 0.1% TFA). Eluted compounds were detected at 218 and 254 nm and lyophilized from water. Melting points were determined on a Boetius block and are uncorrected. ^1^H and ^13^C NMR spectra were measured on a Bruker AVANCE‐600 spectrometer (Billerica, MA, USA; ^1^H at 600.13 MHz, ^13^C at 150.9 MHz) in CDCl_3_, DMSO‐*d*_6_, CD_3_OD or D_2_O solution at 300 K. The 2D‐H,H‐COSY, 2D‐H,C‐HSQC and 2D‐H,C‐HMBC spectra were recorded and used for the structural assignment of proton and carbon signals. IR spectra were recorded on Bruker IFS 55 Equinox apparatus. HRMS spectra were obtained on an FTMS mass spectrometer LTQ‐orbitrap XL (Thermo Fisher, Bremen, Germany) in electrospray ionization mode.

Experimental procedures and analytical data for compounds **3**–**5** are provided in the supporting information.

### *tert*‐Butyl 2‐(*S*)‐(9‐fluorenylmethyloxycarbonylamino)‐3‐bromopropanoate **6**

Ester **5** (14.6 g; 38.1 mmol) and CBr_4_ (15.2 g; 45.7 mmol) were dissolved in 100 ml of DCM (CAS 75‐09‐2). The flask with the reaction mixture was immersed in an ice cooling bath, and PPh_3_ (12 g; 45.7 mmol) in 100 ml DCM was added dropwise under stirring. Stirring at 0 °C was continued for 1 h and then at room temperature (RT) overnight. DCM was removed under reduced pressure, and a brown residue was purified by flash chromatography on silica gel, using a linear gradient of ethyl acetate in petroleum ether. The product was a colorless oil, which was triturated in petroleum ether at −20 °C. Yield 14.2 g (84%). White solid. m.p. 75–76 °C. *R*_f_ = 0.67 (toluene–ethyl acetate 90 : 10). [*α*] 20D = +19.3 (*c* = 0.980; CHCl_3_). ^1^H NMR (600 MHz, DMSO): 1.41 (9H, s, (CH_3_)_3_), 3.65 (1H, dd, *J* = 10.5 and 7.8, –C**Ha**Hb–Br), 3.75 (1H, dd, *J* = 10.5 and 4.5, –CHa**Hb**–Br), 4.24 (1H, t, *J ~* 7.0, >CH–), 4.33 (2H, m, –O–CH_2_–), 4.34 (1H, ddd, *J* = 8.0, 7.8 and 4.5, >CH–Ν), 7.89 (1H, d, *J* = 8.0, –NH–CO), 7.33 (2H, m, Ar–H), 7.42 (2H, m, Ar–H), 7.74 (2H, m, Ar–H), 7.89 (2H, m, Ar–H). ^13^C NMR (150.9 MHz, DMSO): 27.73 ((CH_3_)_3_), 32.81 (–CH_2_‐Br), 46.77 (>CH–), 56.06 (>CH–Ν), 66.06 (–CH_2_–O–CO), 81.99 (O–**C**(CH_3_)_3_), 120.30(2), 125.44(2), 127.25(2) and 127.84(2) (8× Ar=CH–), 140.92(2) and 143.90(2) (4× Ar>C=), 156.04 (N–CO–Ο), 168.15 (O–CO–). IR (KBr) *ν*_max_ (cm^−1^) 3349 m (NH); 1741 vs (C=O) ester; 1696 vs C=O (carbamate); 1514 s (amide II); 1247 s, 1159 s (C–O–C); 3066 w, 3041 w, 1478 m, 760 s, 739 s (ring); 2978 m, 1394 m, 1370 m (CH_3_). HRMS (ESI) calc for C_22_H_24_O_4_NBrNa [M + Na]^+^ 468.07809, found: 468.07840.

### *tert*‐Butyl 2‐(*S*)‐(*tert*‐butoxycarbonylamino)‐3‐hydroxypropanoate **8**

Z‐l‐Ser‐O*t*Bu **4** (12.8 g; 43.3 mmol) was put into a glass pressure bottle and dissolved in 300 ml of methanol, and then 500 mg of 10% Pd/C was added. The mixture was vigorously stirred and allowed to react under the atmosphere of hydrogen (15 psi) at RT overnight. TLC analysis revealed (toluene–ethyl acetate 50 : 50) that the starting compound had completely disappeared. The catalyst was filtered off through celite, and the filter cake was washed in 300 ml of methanol. The filtrate was evaporated *in vacuo* to give 7.2 g of light brown residue, which was immediately dissolved in a 100 ml saturated solution of NaHCO_3_. The flask was placed into the ice bath, and di‐*tert*‐butyl dicarbonate (Boc_2_O) (9.5 g; 43.3 mmol) in 100 ml dioxane was added dropwise under stirring. After the addition of all Boc_2_O, stirring was continued for 1 h at 0 °C and then overnight at RT. Thereafter, 500 ml of water was poured in, and the reaction mixture was transferred to the separatory funnel and extracted four times with 150 ml of ethyl acetate. Combined organic layers were washed consecutively twice with 100 ml of water and twice with 100 ml of brine and dried over anhydrous Na_2_SO_4_. Evaporation of the filtrate under reduced pressure furnished a colorless oil, which was triturated in petroleum ether at −20 °C: colorless solid, yield 9.7 g (86% over two steps). The spectra and physicochemical characteristics of the product are the same as those for **3** prepared by a direct alkylation of Boc‐l‐Ser.

Alternatively, l‐serine **1** (10.5 g; 0.1 mol; [α] 20D = +13, *c* = 5, 5 m HCl) was placed into a 1 l, round‐bottom flask equipped with a magnetic spin bar. The compound **1** was dissolved in the solution of sodium hydrogen carbonate (16.8 g; 0.2 mol) in 150 ml of water. The flask was immersed into the ice cooling bath, and 1.1 eq. of Boc_2_O (24 g; 0.11 mol) in 100 ml of dioxane was added dropwise under vigorous stirring for 30 min. When the addition of Boc anhydride was completed, the reaction mixture was allowed to react for 1 h at 0 °C and then overnight at RT. A saturated aqueous solution of citric acid was added carefully until acidic pH ≈ 3 was reached. The aqueous–organic solution was saturated with sodium chloride, followed by four extractions with ethyl acetate (each with 200 ml). Combined organic phases were washed four times with 100 ml of brine and dried over Na_2_SO_4_. The filtration of the drying agent, followed by evaporation of the filtrate under reduced pressure, furnished 25 g of the crude [(*S*)‐2‐(*tert*‐butoxycarbonylamino)]‐3‐hydroxypropanoic acid as a colorless oil (*R*_f_ = 0.46 ethyl acetate–acetone–methanol–water 6 : 1: 1 : 0.5), which was used in the next step without additional purification.

Compound **8** was prepared in the same manner as **4** by the reaction of 25 g of Boc‐l‐Ser, 200 ml of *tert*‐butyl bromide, potassium carbonate (69.1 g; 0.5 mol) and benzyl triethylammonium chloride (CAS 56‐37‐1, 11.4 g; 0.05 mol) in 450 ml of dimethylacetamide. The required product was a clear oil, which solidified upon standing in a refrigerator at 5 °C. An analytical sample was prepared by trituration in petroleum ether at −20 °C. Yield 18.7 g (72% over two steps), m.p. 77–80 °C. *R*_f_ = 0.55 (toluene–ethyl acetate 50 : 50). [*α*] 20D = −22.2 (*c* = 1.928; EtOH). ^1^H NMR (600 MHz, DMSO): 1.38 (9H, s, (CH_3_)_3_), 1.39 (9H, s, (CH_3_)_3_), 3.60 (2H, dd, *J* = 6.1 and 5.0, –O–CH_2_–), 3.89 (1H, dt, *J* = 8.2, 5.0 and 5.0, >CH–Ν), 4.78 (1H, t, *J* = 6.1, –OH), 6.75 (1H, d, *J* = 8.2, –NH–CO). ^13^C NMR (150.9 MHz, DMSO): 27.87 ((CH_3_)_3_), 28.33 ((CH_3_)_3_), 57.12 (>CH–Ν); 61.63 (–CH_2_–O), 78.32 (O–**C**(CH_3_)_3_), 80.53 (O–**C**(CH_3_)_3_), 155.49 (N–CO–Ο), 170.23 (O–CO–). IR (KBr) *ν*_max_ cm^−1^ 3322 s, 3280 s (NH); 1741 vs (C=O) ester; 1684 vs (C=O) carbamate; 1498 m (amide II); 1155 vs (OC(CH_3_)_3_); 2976 s, 2933 s, 1395 s, 1366 s (CH_3_); 1081 s, 1059 s, 1048 s (C–OH). HRMS (ESI) calc for C_12_H_24_O_5_N [M + H]^+^ 262.16490, found: 262.16507.

### *tert*‐Butyl 2‐(*R*)‐(*tert*‐butoxycarbonylamino)‐3‐hydroxypropanoate **9**

With the method described for **8**, the title enantiomer **9** was prepared from d‐serine (10.5 g; 0.1 mol; [*α*] 20D = −14.75, *c* = 10; 2 N HCl). Yield 17.5 g (67% over two steps), m.p. 76–78 °C. *R*_f_ = 0.55 (toluene–ethyl acetate 50 : 50). [*α*] 20D = +21.6 (*c* = 0.283; EtOH). ^1^H NMR (600 MHz, DMSO): 1.38 (9H, s, (CH_3_)_3_), 1.39 (9H, s, (CH_3_)_3_), 3.60 (2H, dd, *J* = 6.0 and 5.0, HO–C**H**_2_–), 3.89 (1H, dt, *J* = 8.2, 5.0 and 5.0, >CH–Ν), 4.78 (1H, t, *J* = 6.0, –OH), 6.76 (1H, d, *J* = 8.2, –NH–CO). ^13^C NMR (150.9 MHz, DMSO): 27.86 ((CH_3_)_3_), 28.33 ((CH_3_)_3_), 57.12 (>CH–Ν), 61.63 (–CH_2_–O), 78.31 (O–**C**(CH_3_)_3_), 80.52 (O–**C**(CH_3_)_3_), 155.48 (N–CO–Ο), 170.22 (O–CO–). IR (KBr) *ν*_max_ (cm^−1^) 3321 m, 3278 m (NH); 1740 vs (C=O) ester; 1683 vs (C=O) carbamate; 1497 m (amide II); 1155 vs (C–O); 2975 m, 2933 s, 1395 s, 1366 s (CH_3_); 1153 vs (OC(CH_3_)_3_); 1081 s, 1059 s, 1048 s (C–OH). HRMS (ESI) calc for C_12_H_24_O_5_N [M + Na]^+^ 284.14684, found: 284.14692.

*tert*‐Butyl 2‐(*tert*‐butoxycarbonylamino)acrylate **10**, *tert*‐Butyl 2‐(*S*)‐(*tert*‐butoxycarbonylamino)‐3‐azidopropanoate **11** and *tert*‐Butyl 2‐(*R*)‐(*tert*‐butoxycarbonylamino)‐3‐azidopropanoate **12**

Ester **8** (18.7 g; 71.6 mmol) was dissolved in 150 ml of DCM with TEA (10.9 g; 107.4 mmol). The reaction mixture was cooled in the ice bath, and MsCl (9 g; 78.8 mmol) was added dropwise. After half an hour of cooling and stirring, TLC analysis revealed that starting compound **8** had been consumed. Except for mesyl intermediate (*R*_f_ = 0.73 in toluene–ethyl acetate, 80 : 20), the elimination product **10** (*R*_f_ = 0.88 in toluene–ethyl acetate, 80 : 20) was also detected on the TLC plate. Excess of the base was eliminated by the addition of 1 m solution of citric acid; the lower organic layer was washed with 50 ml of water and 50 ml of brine and dried over Na_2_SO_4_. Volatile materials were evaporated under reduced pressure to give 23.8 g of brown oil. The crude intermediate was dissolved in 100 ml DMSO with NaN_3_ (9.1 g; 140.4 mmol) and heated at 70 °C overnight. After cooling, 200 ml of water was added, and the solution was extracted four times with 200 ml of diethyl ether. The combined organic layers were washed twice with 100 ml of water and twice with 100 ml brine and dried over Na_2_SO_4_. The drying agent was filtered off, and the filtrate was evaporated under reduced pressure to afford 20 g of yellow residue, which was purified by flash chromatography on silica gel, using a linear gradient of diethyl ether in petroleum ether. Two major compounds were isolated, the product of elimination **10** and the required azide **11**. Yield **10** 2.7 g (16% over two steps). Yield **11** 9.7 g (47% over two steps).

Alternatively, ester **8** (7.3 g; 27.9 mmol) was treated in 50 ml DCM with (3.3 g; 41.9 mmol) pyridine and (3.5 g; 30.7 mmol) MsCl, followed by the addition of NaN_3_ (3.5 g; 53.1 mmol) in 50 ml DMSO by the protocol described earlier. The starting compound was fully reacted after 24 h; the formation of **10** during the mesylation step was also observed. Yield **10** 1.8 g (26% over two steps), yield **11** 3.7 g (46% over two steps).

Alternatively, **13** (9.7 g; 29.9 mmol) was dissolved in 50 ml of DMSO with NaN_3_ (3.9 g; 59.8 mmol) and heated at 70 °C overnight. After cooling, 100 ml of water was added, and the solution was extracted four times with 50 ml of diethyl ether. The combined organic layers were washed twice with 50 ml of water and twice with 50 ml of brine and dried over Na_2_SO_4_. The drying agent was filtered off, and the filtrate was evaporated under reduced pressure to afford 8.6 g of dark yellow residue, which was purified by flash chromatography on silica gel, using a linear gradient of diethyl ether in petroleum ether. Yield **10** 2.9 g (40%), yield **11** 3.5 g (41%).

Using the method described for **11**, the title enantiomer **12** was prepared from **9** (10.5 g; 40.2 mmol), MsCl (5.1 g; 44.2 mmol), TEA (6.1 g; 60.3 mmol) and NaN_3_ (5.3 g; 80.4 mmol). Yield **10** 2.1 g (21%), yield **12** 5.8 g (50%).

Compound **10**. Colorless oil. *R*_f_ = 0.77 (toluene–ethyl acetate 90 : 10). ^1^H NMR (600 MHz, CDCl_3_): 1.47 (9H, s, (CH_3_)_3_), 1.51 (9H, s, (CH_3_)_3_), 5.63 and 6.07 (2H, 2× d, *J* = 1.5, =CH_2_), 7.03 (1H, br s, –NH–CO). ^13^C NMR (150.9 MHz, CDCl_3_): 27.88 ((CH_3_)_3_), 28.22 ((CH_3_)_3_), 80.39 (O–**C**(CH_3_)_3_), 82.54 (O–**C**(CH_3_)_3_), 103.95 (=CH_2_), 132.43 (=C<), 152.60 (N–CO–Ο), 163.02 (O‐CO‐). IR (CCl_4_) *ν*_max_ (cm^−1^) 3461 w, 3421 m (NH); 1736 vs (C=O) ester; 1707 vs (C=O) carbamate; 1509 vs (amide II); 1157 vs, 1065 vs (C–O–C); 2980 s, 2934 s, 1393 s, 1369 s (CH_3_); 3005 w, 1641 m (C=C). HRMS (ESI) calc for C_12_H_21_O_4_NNa [M + Na]^+^ 266.13628, found: 266.13635.

Compound **11**. Colorless liquid. *R*_f_ = 0.59 (toluene–ethyl acetate 90 : 10). [*α*] 20D = +26.9 (*c* = 0.304; CHCl_3_). ^1^H NMR (600 MHz, CDCl_3_): 1.36 (9H, s, (CH_3_)_3_), 1.40 (9H, s, (CH_3_)_3_), 3.57 (1H, dd, *J* = 12.4 and 3.7, –C**Ha**Hb–Ν_3_), 3.62 (1H, dd, *J* = 12.4 and 3.5, –CHa**Hb**–Ν_3_), 4.25 (1H, ddd, *J* = 7.4, 3.7 and 3.5, >CH–Ν), 5.26 (1H, d, *J* = 7.4, –NH–CO).^13^C NMR (150.9 MHz, CDCl_3_): 27.92 ((CH_3_)_3_), 28.27 ((CH_3_)_3_), 53.00 (–CH_2_–N_3_), 54.13 (>CH–Ν); 80.19 (O–**C**(CH_3_)_3_), 83.13 (O–**C**(CH_3_)_3_), 155.08 (N–CO–Ο), 168.72 (O–CO–). IR (CCl_4_) *ν*_max_ (cm^−1^) 3435 m, 3364 m (NH); 2108 vvs (N_3_); 1740 vs (C=O) ester; 1719 vs (C=O) carbamate; 1498 s (amide II); 1155 vs (C(OCH_3_)_3_); 2980 s, 2935 s, 1394 s, 1369 s (CH_3_). HRMS (ESI) calc for C_12_H_22_O_4_N_4_Na [M + Na]^+^ 309.15333, found: 309.15336.

Compound **12**. Colorless liquid. *R*_f_ = 0.59 (toluene–ethyl acetate 90 : 10). [*α*] 20D = −27.9 (*c* = 0.369; CHCl_3_). ^1^H NMR (600 MHz, CDCl_3_): 1.44 (9H, s, (CH_3_)_3_), 1.48 (9H, s, (CH_3_)_3_), 3.65 (1H, dd, *J* = 12.3 and 3.6, –C**Ha**Hb–N_3_), 3.70 (1H, dd, *J* = 12.3 and 3.6, –CHa**Hb**–N_3_), 4.33 (1H, dt, *J* = 7.3, 3.6 and 3.6, >CH–Ν), 5.35 (1H, br d, *J* = 7.3, –NH–CO). ^13^C NMR (150.9 MHz, CDCl_3_): 27.91 ((CH_3_)_3_), 28.26 ((CH_3_)_3_), 52.99 (–CH_2_–N_3_), 54.13 (>CH–Ν), 80.17 (O–**C**(CH_3_)_3_), 83.11 (O–**C**(CH_3_)_3_), 155.08 (N–CO–Ο), 168.71 (O–CO–). IR (CCl_4_) *ν*_max_ (cm^−1^) 3435 m (NH); 2109 vvs (N_3_); 1741 vs (C=O) ester; 1716 vs (C=O) carbamate; 1492 s (amide II); 1155 vs (C(OCH_3_)_3_); 2981 s, 2933 s, 1393 s, 1369 s (CH_3_). HRMS (ESI) calc for C_12_H_22_O_4_N_4_Na [M + Na]^+^ 309.15333, found: 309.15342.

### *tert*‐Butyl 2‐(*S*)‐(*tert*‐butoxycarbonylamino)‐3‐bromopropanoate **13**

Ester **8** (11.5 g; 44 mmol) and CBr_4_ (17.5 g; 52.8 mmol) were dissolved in 100 ml of DCM. The flask with the reaction mixture was immersed in the ice cooling bath, and PPh_3_ (13.8 g; 52.8 mmol) in 50 ml of DCM was added dropwise under stirring. Stirring was continued for 1 h at 0 °C and then at RT overnight. DCM was removed under reduced pressure, and the brown residue was purified by flash chromatography on silica gel, using a linear gradient of ethyl acetate in petroleum ether. The product was a colorless oil, which was triturated in petroleum ether at −20 °C. Yield 9.7 g (68%). White solid, m.p. 64–65 °C. *R*_f_ = 0.65 (toluene–ethyl acetate 90 : 10). [*α*] 20D = −7 (*c* = 0.284; CHCl_3_). ^1^H NMR (600 MHz, DMSO): 1.39 (9H, s, (CH_3_)_3_), 1.41 (9H, s, (CH_3_)_3_), 3.61 (1H, dd, *J* = 10.4 and 7.8, –C**Ha**Hb–Br), 3.70 (1H, dd, *J* = 10.4 and 4.5, –CHa**Hb**–Br), 4.22 (1H, ddd, *J* = 8.2, 7.8 and 4.5, >CH–Ν), 7.21 (1H, d, *J* = 8.2, –NH–CO). ^13^C NMR (150.9 MHz, DMSO): 27.72 ((CH_3_)_3_), 28.27 ((CH_3_)_3_), 32.89 (–CH_2_–Br), 55.75 (>CH–Ν); 78.77 (O–**C**(CH_3_)_3_), 81.75 (O–**C**(CH_3_)_3_), 155.29 (N–CO–Ο), 168.41 (O–CO–). IR (KBr) *ν*_max_ (cm^−1^) 3433 m (NH); 1736 vs (C=O) ester; 1714 vs (C=O) carbamate; 1498 s (amide II); 1157 s, 1067 s (C–O–C); 2980 s, 2934 s, 1395 s, 1369 s (CH_3_). HRMS (ESI) calc for C_12_H_22_O_4_NBrNa [M + Na]^+^ 346.06244, found: 346.06254.

2‐(*S*)‐(9‐Fluorenylmethyloxycarbonylamino)‐3‐azidopropanoic acid (Fmoc‐*β*‐azido‐l‐Ala) **14** Compound **11** (9.6 g; 33.5 mmol) was treated with the cleavage cocktail, consisting of 18.8 ml of DCM, 18.8 ml of TFA and 2.4 ml of water. The reaction started with severe liberation of CO_2_ and isobutene and continued at RT overnight under stirring. Volatile materials were removed on the rotary evaporator. The yellow residue was dissolved in a solution of NaHCO_3_ (11.3 g; 134 mmol) in 50 ml of water. The reaction mixture was cooled in the ice bath, and Fmoc‐OSu (11.3 g; 33.5 mmol) in 50 ml dioxane was added dropwise under vigorous stirring. The reaction mixture was allowed to react for 1 h at 0 °C and then overnight at RT. The flask was again ice cooled, and concentrated hydrochloric acid was carefully added until acidic pH ≈ 1 was reached. The reaction mixture was extracted thrice with 100 ml of ethyl acetate. Thereafter, the combined organic layers were successively washed once with 100 ml of water and twice with 100 ml of brine, followed by drying on Na_2_SO_4_. Evaporation of the filtrate gave a brown oil, which was purified by flash chromatography on silica gel, using a linear gradient of 1% CH_3_COOH/ethyl acetate in toluene. Evaporation of the product afforded a yellow semisolid, which was triturated in toluene at −20 °C. Yield 10.2 g (86%). Colorless solid. m.p. 119–120 °C. *R*_f_ = 0.63 (ethyl acetate–acetone–methanol–water 6 : 1 : 1 : 0.5). [*α*] 20D = −4.8 (*c* = 0.271; DMF). ^1^H NMR (600 MHz, DMSO): 3.53 (1H, dd, *J* = 12.1 and 6.4, –C**Ha**Hb–N_3_), 3.68 (1H, dd, *J* = 12.1 and 3.6, –CHa**Hb**–N_3_), 3.98 (1H, ddd, *J* = 7.0, 6.4 and 3.6, >CH–Ν), 4.22 (1H, m, >CH–), 4.22 and 4.33 (2H, 2× m, –O–CH_2_–), 6.95 (1H, d, *J* = 7.0, –NH–CO), 7.31 (2H, m, Ar–H), 7.40 (2H, m, Ar–H), 7.69 (2H, m, Ar–H), 7.87 (2H, m, Ar–H). ^13^C NMR (150.9 MHz, DMSO): 46.92 (>CH–), 52.89 (–CH_2_–N_3_), 56.08 (>CH–Ν), 65.86 (–CH_2_–O–CO), 120.30(2), 125.40, 125.46, 127.29(2) and 127.82(2) (8× Ar=CH–), 140.90(2), 144.01 and 144.10 (4× Ar>C=), 155.83 (N–CO–Ο), 172.29 (–COOH). IR (KBr) *ν*_max_ (cm^−1^) 3402 m (NH); 2108 vs (N_3_); 1719 vs (C=O) acid; 1693 vs (C=O) carbamate; 1539 s (amide II); 3065 w, 3041 w, 1603 vs, 1478 s, 1451 s, 757 m, 740 s (ring). HRMS (ESI) calc for C_18_H_15_O_4_N_4_[M − H]^+^ 351.10988, found: 351.10968.

Alternatively, compound **14** was prepared from **27**. Triflic anhydride (13.3 g; 47.2 mmol) was added dropwise under ice cooling and vigorous stirring to the two‐phase system of NaN_3_ (15.3 g; 236 mmol) in 60 ml of water and 70 ml of DCM. The ice bath was removed and stirring continued for 2 h. The aqueous layer was separated and extracted twice with 50 ml of DCM. Thereafter, the combined organic phases were washed with 5% NaHCO_3_. The resulting solution of triflic azide (TfN_3_) in DCM was immediately added dropwise to the suspension of **27** (7.7 g; 23.6 mmol), NaHCO_3_ (19.8 g; 236 mmol) and CuSO_4_·5H_2_O (60 mg; 23.6 μmol) in 50 ml of water and 150 ml of methanol, and the mixture was stirred at RT overnight. Volatile material was evaporated, and the remaining slurry was carefully acidified with concentrated HCl until pH 1–2 was reached. The reaction mixture was extracted four times with 100 ml of ethyl acetate. The combined organic layers were washed twice with 100 ml of water and twice with 100 ml of brine and dried over Na_2_SO_4_. Filtering off the drying agent and evaporation of the filtrate gave 8 g of brown residue, which was purified by flash chromatography on silica gel, using a linear gradient of 1% CH_3_COOH/ethyl acetate in toluene. The yellow oil was triturated in toluene at −20 °C to give the pure product. Yield 6.6 g (80%). Physicochemical characteristics were consistent with the above‐listed ones.

Alternatively, compound **14** was prepared from **32**. Compound **32** (8.4 g; 36.5 mmol) was treated with a mixture of 18.8 ml of DCM, 18.8 ml of TFA and 2.4 ml of water. After 2 h of stirring, volatile materials were evaporated, and the yellow oil was dissolved in 50 ml of water with NaHCO_3_ (9.2 g; 109.5 mmol). The flask was immersed in an ice cooling bath, and Fmoc‐OSu (12.3 g; 36.5 mol) in 100 ml of dioxane was added dropwise during a period 15 min under vigorous stirring. When the addition of Fmoc‐OSu was complete, the slurry was allowed to react for 1 h at 0 °C and then overnight at RT. The reaction mixture was again cooled in an ice bath, and concentrated HCl was added dropwise until pH ~ 0–1 was reached. Thereafter, 150 ml of water was added, and the reaction mixture was extracted thrice with 100 ml of ethyl acetate. The combined organic layers were washed once with 100 ml of water and twice with 100 ml of brine and dried over Na_2_SO_4_. The filtrate was evaporated, and the resulting brown oil was subjected to flash chromatography on silica gel, using a linear gradient of 1% CH_3_COOH/ethyl acetate in toluene. The yellow oil was triturated in toluene at −20 °C to afford the pure product. Yield 8.2 g (64%). Physicochemical characteristics were consistent with the above‐listed ones.

### 2‐(*R*)‐(9‐Fluorenylmethyloxycarbonylamino)‐3‐azidopropanoic Acid (Fmoc‐*β*‐azido‐d‐Ala) **15**

With the method described for **14**, the title enantiomer **15** was prepared from **12** (7.4 g; 25.9 mmol), 18.8 ml TFA, NaHCO_3_ (8.7 g; 103.6 mmol) and Fmoc‐OSu (8.7 g; 25.9 mmol). Yield 7.8 g (86%). Colorless solid. m.p. 117–119 °C. *R*_f_ = 0.63 (ethyl acetate–acetone–methanol–water 6 : 1 : 1 : 0.5). [*α*] 20D = +5.1 (*c* = 0.235; DMF). ^1^H NMR (600 MHz, DMSO): 3.61 (1H, dd, *J* = 13.0 and 5.2, –C**Ha**Hb–N_3_), 3.64 (1H, dd, *J* = 13.0 and 7.1, –CHa**Hb**–N_3_), 4.24 (1H, ddd, *J* = 8.3, 7.1 and 5.2, >CH–Ν), 4.24 (1H, m, >CH–), 4.31 (1H, dd, *J* = 10.5 and 6.7, –C**Ha**Hb–O), 4.33 (1H, dd, *J* = 10.5 and 7.5, –CHa**Hb**–O), 7.33 (2H, m, Ar–H), 7.42 (2H, m, Ar–H), 7.74 (2H, m, Ar–H), 7.89 (2H, m, Ar–H), 7.92 (1H, d, *J* = 8.3, –NH–CO). ^13^C NMR (150.9 MHz, DMSO): 46.79 (>CH–), 51.12 (–CH_2_–N_3_), 54.01 (>CH–Ν), 66.03 (–CH_2_–O–CO), 120.29(2), 125.45(2), 127.26(2) and 127.84(2) (8× Ar=CH–), 140.90(2), 143.92 and 143.96 (4× Ar>C=), 156.22 (N–CO–Ο), 171.23 (COOH). IR (KBr) *ν*_max_ (cm^−1^) 3403 m, 3305 m (NH); 2109 vs (N_3_); 1712 vs (C=O) acid; 1707 vs (C=O) carbamate; 1538 s (amide II); 3065 w, 3041 w, 1611 w, 1580 w, 1478 m, 1451 s, 758 m, 740 s (ring). HRMS (ESI) calc for C_18_H_16_O_4_N_4_Na[M + Na]^+^ 375.10638, found: 375.10653.

### 2‐(*S*)‐Amino‐3‐azidopropanoic Acid Trifluoroacetic Acid Salt **16**

One gram of 2‐Cl‐Trt‐chloride resin (Merck Novabiochem, Darmstadt, Germany, capacity 1.5 mmol/g, 100–200 mesh) was placed in a 20 ml syringe with a frit and preswollen in 10 ml DMF for half an hour. The solvent was removed, and **14** (0.575 g; 1.5 mmol) in 4 ml of DMF and DIPEA (783 μl; 4.5 mmol) in 2 ml of DMF were added. The syringe was agitated by shaking for 1.5 h, followed by washing (3× 10 ml of DMF). The reaction was terminated by two subsequent additions of a mixture of 5.1 ml of DCM, 0.6 ml of CH_3_OH and 0.3 ml of DIPEA, each for 5 min. The resin was washed thrice with 10 ml of DCM and thrice with 10 ml of DMF. The Fmoc group was cleaved with 20% (*v*/*v*) piperidine in DMF (10 ml for 5 and 30 min). The resin was washed thrice with 10 ml of DMF and thrice with 10 ml of DCM. Finally, the product was cleaved from the resin by three subsequent treatments with a mixture of 2 ml of AcOH, 2 ml of trifluoroethanol, CAS 75‐89‐8, and 6 ml of DCM, each for 15 min. Filtrates were evaporated to dryness, and the crude material was subjected to RP‐HPLC. The following gradient was used: *t* = 0 min (2% B), *t* = 15 min (15% B), *t* = 31 min (100% B). Yield 270 mg (74%). White lyophilisate. [*α*] 20D = +21.8 (*c* = 0.262; H_2_O). ^1^H NMR (600 MHz, D_2_O + NaOD): 3.51 (1H, dd, *J* = 5.6 and 4.3, >CH–N), 3.60 (1H, dd, *J* = 12.6 and 4.3, –C**Ha**Hb–N_3_), 3.65 (1H, dd, *J* = 12.6 and 5.6, –CHa**Hb**–N_3_). IR (KBr) *ν*_max_ (cm^−1^) 2122 vvs (N_3_); 1733 s (C=O) acid; 1640 s, 1443 m (C=O) CF_3_COO^−^; 1619 m, 1535 m (NH_3_^+^); 1207 s, 1151 m (CF). HRMS (ESI) calc for C_3_H_7_O_2_N_4_[M + H]^+^ 131.05635, found: 131.05640.

### Fmoc‐*β*‐azido‐Ala‐Val‐OBn **18**

Fmoc‐*β*‐azido‐Ala **14** (326 mg; 1 mmol), l‐Val‐OBn·TsOH (379 mg; 1 mmol), HOBt·H_2_O (135 mg; 1 mmol), bromotripyrrolidinophosphonium hexafluorophosphate (CAS 132705‐51‐2, 606 mg; 1.3 mmol) and DIPEA (387 mg; 3 mmol) were stirred at RT in 7 ml of DMF. The solvent was evaporated under reduced pressure, and the residue was purified by flash chromatography on silica gel, using a linear gradient of ethyl acetate in toluene. Yield 400 mg (74%). Semisolid. *R*_f_ = 0.54 (toluene–ethyl acetate 80 : 20). [*α*] 20D = −7.4 (*c* = 0.270; DMF). ^1^H NMR (600 MHz, DMSO): 0.862 (3H, d, *J* = 6.8, –CH_3_), 0.866 (3H, d, *J* = 6.8, –CH_3_), 2.08 (1H, m, –CH<), 3.39 (1H, dd, *J* = 12.5 and 9.1, –C**Ha**Hb–N_3_), 3.47 (1H, dd, *J* = 12.5 and 4.4, –CHa**Hb**–N_3_), 4.22–4.31 (3H, m, >CH–CH_2_–O–CO), 4.24 (1H, t, *J* = 8.2, >CH–Ν), 4.43 (1H, ddd, *J* = 9.1, 8.7 and 4.4, >CH–Ν), 5.10 and 5.15 (2H, 2× d, *J* = 12.4, –O–CH_2_–), 7.32 (2H, m, Ar–H), 7.33–7.37 (5H, m, –C_6_H_5_), 7.42 (2H, m, Ar–H), 7.73 (2H, m, Ar–H), 7.89 (2H, m, Ar–H). 7.81 (1H, d, *J* = 8.7, –NH–CO), 8.36 (1H, d, *J* = 8.2, –NH–CO).^13^C NMR (150.9 MHz, DMSO): 18.21 (CH_3_), 19.06 (CH_3_), 27.81 ((CH_3_)_3_), 30.02 (>CH–), 46.78 (>CH–), 51.69 (–CH_2_–N_3_), 54.43 (>CH–Ν), 57.79 (>CH–Ν), 66.04 (–CH_2_–O–CO), 66.22 (–CH_2_–O–CO), 120.29(2), 125.48, 125.51, 127.25(2) and 127.84(2) (8× Ar=CH–), 128.20–128.60(5) (–C_6_H_5_), 140.90(2), 143.91 and 143.97 (4× Ar>C=), 156.10 (N–CO–Ο), 169.65 (N–CO–), 171.17 (N–CO–). IR (KBr) *ν*_max_ (cm^−1^) 3291 vs (NH); 2105 vs (N_3_); 1733 vs (C=O) ester; 1690 vs (C=O) carbamate; 1655 vs (C=O) amide; 1539 vs (amide II); 1252 s (C–O–C) ester; 3066 w, 3038 w, 1603 vs, 1478 m 1464 m, 1450 s, 758 s, 740 s (ring). HRMS (ESI) calc for C_30_H_31_O_5_N_5_Na[M + Na]^+^ 564.22174, found: 564.22118.

Experimental procedures and analytical data for compound **20** are provided in the supporting information.

### Fmoc‐*β*‐azido‐Ala‐Val‐O*t*Bu **21**

Fmoc‐*β*‐azido‐Ala **14** (0.652 g; 2 mmol), l‐Val‐O*t*Bu·TsOH **20** (0.419 g; 2 mmol), bromotripyrrolidinophosphonium hexafluorophosphate (1.17 g; 2.5 mmol) and DIPEA (1 g; 8 mmol) in 10 ml of DMF were stirred overnight at RT. The solvent was evaporated under reduced pressure, and the residue was purified by flash chromatography on silica gel, using a linear gradient of ethyl acetate in toluene. An analytical sample was gained by crystallization from a mixture of ethyl acetate–petroleum ether. Yield 700 mg (70%). *R*_f_ = 0.82 (toluene–ethyl acetate 50 : 50). White solid, 154–155 °C. [*α*] 20D = −4.2 (*c* = 0.357; DMF). ^1^H NMR (600 MHz, DMSO): 0.87 (3H, d, *J* = 6.9, CH_3_), 0.88 (3H, d, *J* = 6.9, CH_3_), 1.39 (9H, s, (CH_3_)_3_), 2.04 (1H, m, >CH–), 1.41 (9H, s, (CH_3_)_3_), 3.44 (1H, dd, *J* = 12.5 and 8.8, –C**Ha**Hb–N_3_), 3.54 (1H, dd, *J* = 12.5 and 4.2, –CHa**Hb**–N_3_), 4.23 (1H, dd, *J* = 7.5 and 6.9, >CH–), 4.27 (1H, dd, *J* = 10.4 and 6.9, –C**Ha**Hb–O–CO), 4.31 (1H, dd, *J* = 10.4 and 7.5, –CHa**Hb**–O–CO), 4.44 (1H, td, *J* = 8.8 and 4.2, >CH–Ν), 7.84 (1H, d, *J* = 8.8, –NH–CO), 8.20 (1H, d, *J* = 8.2, –NH–CO), 7.32 (2H, m, Ar–H), 7.41 (2H, m, Ar–H), 7.73 (2H, m, Ar–H), 7.89 (2H, m, Ar–H). ^13^C NMR (150.9 MHz, DMSO): 18.90 (CH_3_), 19.10 (CH_3_), 27.81 ((CH_3_)_3_), 30.13 (>CH–), 46.79 (>CH–), 51.81 (–CH_2_–N_3_), 54.49 (>CH–N), 58.19 (>CH–N), 66.08 (–CH_2_–O–CO), 80.95 (O–**C**(CH_3_)_3_), 120.33(2), 125.52(2), 127.28(2) and 127.87(2) (8× Ar=CH–), 140.92(2), 143.94 and 143.99 (4× Ar>C=), 156.14 (N–CO–Ο), 169.45 (N–CO–), 170.43 (–O–CO–). IR (KBr) *ν*_max_ (cm^−1^) 3335 s, 3266 s (NH); 2964 s, 2873 w, 1479 m, 1386 s, 1370 s (CH_3_); 2106 vs (N_3_); 1730 vs (C=O) ester; 1714 vs (C=O) carbamate; 1659 vs (C=O) amide; 1555 s, 1533 vs (amide II); 1253 vs (C–O); 1160 s (–C(CH_3_)_3_); 1143 s (–CH(CH_3_)_2_); 3069 w, 3045 w, 3007 w, 1479 m, 1451 s, 1312 s, 1110 m, 799 m, 761 s, 742 vs (ring). HRMS (ESI) calc for C_27_H_33_O_5_N_5_Na[M + Na]^+^ 530.23739, found: 530.23732.

### *β*‐Azido‐Ala‐Val **22**

Compound **21** (600 mg; 1.2 mmol) was treated with a mixture consisting of 2 ml of DCM, 2.5 ml of TFA and 40 μl of water. After 2 h, TLC analysis revealed completely deprotected *tert*‐butyl moiety, and the volatile material was evaporated under reduced pressure. 2‐Cl‐Trt‐chloride resin (0.8 g, Merck Novabiochem, capacity 1.5 mmol/g, 100–200 mesh) was placed into a 20 ml syringe with a frit and preswollen in 10 ml of DMF for half an hour. DMF was removed, and the crude acid Fmoc‐*β*‐azido‐Ala‐Val in 4 ml DMF and DIPEA (627 μl; 3.6 mmol) in 2 ml DMF were added. The syringe was agitated by shaking for 1.5 h, followed by washing thrice with 10 ml of DMF. In the next step, the Fmoc group was cleaved with 20% (*v*/*v*) piperidine in DMF (10 ml for 5 and 30 min). The resin was washed thrice with 10 ml of DMF and thrice with 10 ml of DCM. Finally, the product was cleaved from resin by three subsequent treatments with 10 ml of AcOH, 15 min each. Filtrates were combined and evaporated to dryness. The residue was sonicated for 10 min in 15 ml of diethyl ether. The white precipitate was decanted and dissolved in 2 ml of hot acetonitrile (60 °C). After cooling, the crystals were filtered off and washed with 5 ml of diethyl ether. Yield 181 mg (67%). White solid, m.p. 187–189 °C. [*α*] 20D = +8.9 (*c* = 0.357; DMSO). ^1^H NMR (600 MHz, DMSO): 0.856 (3H, d, *J* = 6.8, CH_3_), 0.865 (3H, d, *J* = 6.8, CH_3_), 2.07 (1H, m, >CH–), 3.44 (1H, dd, *J* = 12.4 and 6.4, –C**Ha**Hb–N_3_), 3.48 (1H, dd, *J* = 12.4 and 4.4, –CHa**Hb**–N_3_), 3.55 (1H, dd, *J* = 6.4 and 4.4, >CH–N), 4.13 (1H, dd, *J* = 8.6 and 5.2, >CH–N), 8.17 (1H, d, *J* = 8.6, –NH–CO) ^13^C NMR (150.9 MHz, DMSO): 18.01 (CH_3_), 19.43 (CH_3_), 30.59 (>CH–), 54.34 (>CH–N), 54.49 (–CH_2_–N_3_), 57.46 (>CH–N), 171.53 (N–CO–), 173.16 (–COOH). IR (KBr) *ν*_max_ (cm^−1^) 3433 m + vbr (NH); 2967 s, 2875 m, 1389 s, 1376 m (CH_3_); 2938 m, 1443 m (CH_2_); 2110 vs (N_3_); 1671 vs (C=O) amide; 1506 vs (amide II); 1581 s; 1403 m (COO^−^); 1174 m (–CH(CH_3_)_2_). HRMS (ESI) calc for C_8_H_14_O_3_N_5_[M − H]^−^ 228.11021, found: 228.10999.

Experimental procedures and analytical data for compounds **25** and **26** are provided in the supporting information.

### 2‐(*S*)‐(9‐Fluorenylmethyloxycarbonylamino)‐3‐aminopropionic Acid **27**

Compound **25** (11.1 g; 31.3 mmol) was suspended in a mixture of 48 ml of acetonitrile, 48 ml of ethyl acetate and 24 ml of water. (Diacetoxyiodo)benzene (12.1 g; 37.6 mmol) was added under vigorous stirring in five portions, and the resulting slurry was allowed to react overnight. The flask was ice cooled, and the crystals were filtered off in a Büchner funnel and washed with 100 ml of chilled ethyl acetate. Final purification was carried out by dissolving the solid in 150 ml of hot isopropyl alcohol (70 °C). After cooling, the crystals were filtered off and dried under deep vacuum. Yield 7.7 g (75%). White solid, m.p. 130–132 °C. *R*_f_ = 0.75 (ethyl acetate–acetone–methanol–water 4 : 1 : 1 : 1). [*α*] 20D = +4.6 (*c* = 0.306; acetic acid). ^1^H NMR (600 MHz, DMSO): 2.87 and 3.04 (2H, 2× m, –CH_2_–N), 3.80 (1H, m, >CH–N), 4.20–4.28 (3H, m, >CH–CH_2_–O–CO), 6.91 (1H, br s, –NH–CO), 7.32 (2H, m, Ar–H), 7.40 (2H, m, Ar–H), 7.70 (2H, m, Ar–H), 7.88 (2H, m, Ar–H). ^13^C NMR (150.9 MHz, DMSO): 40.89 (–CH_2_–NH_2_), 46.84 (>CH–), 52.00 (>CH–N), 65.92 (–CH_2_–O–CO), 120.28(2), 125.44, 125.47, 127.29(2) and 127.81(2) (8× Ar=CH–), 140.89(2), 144.04 and 144.10 (4× Ar>C=), 155.91 (N–CO–Ο), 171.56 (–COOH). IR (KBr) *ν*_max_ (cm^−1^) 3409 s (NH); 1703 vs + vbr (C=O) acid and carbamate; 1530 m (amide II); 3065 w, 3040 w, 1479 m, 1450 m, 759 m, 740 s (ring). HRMS (ESI) calc for C_18_H_18_O_4_N_2_Na [M + Na]^+^ 349.11588, found: 349.11595.

### 2‐(*S*)‐(9‐Fluorenylmethyloxycarbonylamino)‐4‐aminobutanoic Acid **28**

Compound **28** was prepared by the reaction of **26** (6.6 g; 17.9 mmol) and PIDA (6.9 g; 21.5 mmol) by the method used previously for **27**. Yield 3.4 g (56%). *R*_f_ = 0.70 (ethyl acetate–acetone–methanol–water 4 : 1 : 1 : 1). [*α*] 20D = +22.4 (*c* = 0.241; DMSO). ^1^H NMR (500 MHz, DMSO): 1.87 (2H, m, –CH_2_–), 2.87 (2H, t, *J* = 6.5, –CH_2_–N), 3.71 (1H, q, *J* = 6.3, >CH–N), 4.20 (1H, m, >CH–), 4.24 (2H, m, –CH_2_–O), 6.76 (1H, d, *J* = 6.3, –NH–CO), 7.32 (2H, m, Ar–H), 7.40 (2H, m, Ar–H), 7.68 (2H, m, Ar–H), 7.87 (2H, m, Ar–H). ^13^C NMR (125.7 MHz, DMSO): 30.97 (–CH_2_–), 37.32 (–CH_2_–N), 46.97 (>CH–), 54.70 (>CH–N), 65.84 (–CH_2_–O–CO), 120.40(2), 125.54(2), 127.42(2) and 127.93(2) (8× Ar=CH–), 140.99(2) and 144.25(2) (4× Ar>C=), 155.70 (N–CO–Ο), 173.64 (–COOH). IR (KBr) *ν*_max_ (cm^−1^) 3384 m (NH); 1727 vs (C=O) acid; 1705 vs (C=O) carbamate; 1518 s (amide II); 3039 w, 3020 w, 1593 s, 1465 m, 1450 m, 1107 m, 1033 m, 760 m, 736 s (ring); 1478 m (CH_2_). HRMS (ESI) calc for C_19_H_21_O_4_N_2_Na [M + Na]^+^ 363.13153, found: 363.13162.

### 2‐(*S*)‐(9‐Fluorenylmethyloxycarbonylamino)‐4‐azidobutanoic Acid **29**

With the protocol previously employed for **11**, the reaction of **28** (4.5 g; 13.2 mmol), NaHCO_3_ (11.1 g; 132 mmol) and CuSO_4_·5H_2_O (32 mg; 132 μmol) in a mixture of 75 ml of methanol and 25 ml of water with TfN_3_ in dichloromethane gave azide **29**. TfN_3_ was prepared by the reaction of NaN_3_ (8.6 g; 132 mmol) and triflic anhydride (7.5 g; 26.4 mmol) in 40 ml of water and 60 ml of DCM. Yield 4.4 g (92%). *R*_f_ = 0.68 (ethyl acetate–acetone–methanol–water 6 : 1: 1: 0.5). [*α*] 20D = −17.3 (*c* = 0.294; CH_3_OH). ^1^H NMR (500 MHz, DMSO): 1.84 and 1.97 (2H, 2× m, –CH_2_–), 3.34 (1H, ddd, *J* = 12.5, 8.2 and 6.5, –C**Ha**Hb–N_3_), 3.44 (1H, dd, *J* = 12.5, 7.1 and 5.3, –CHa**Hb**–N_3_), 4.04 (1H, ddd, *J* = 10.0, 8.2 and 4.4, >CH–N), 4.23 (1H, m, >CH–), 4.32 (2H, m, –CH_2_–O), 7.33 (2H, m, Ar–H), 7.41 (2H, m, Ar–H), 7.70 (1H, d, *J* = 8.2, –NH–CO), 7.71 (2H, m, Ar–H), 7.88 (2H, m, Ar–H). ^13^C NMR (125.7 MHz, DMSO): 30.20 (–CH_2_–), 46.94 (>CH–), 47.85 (–CH_2_–N_3_), 51.50 (>CH–N), 65.91 (–CH_2_–O–CO), 120.42, 120.44, 125.51, 125.53, 127.39(2) and 127.97(2) (8× Ar= CH–), 141.02(2), 144.04 and 144.08 (4× Ar>C=), 156.47 (N–CO–Ο), 173.67 (–COOH). IR (KBr) *ν*_max_ (cm^−1^) 3333 m (NH); 2950 w, 1478 w (CH_2_); 2107 vs (N_3_); 1717 vs (C=O) acid; 1697 vs (C=O) carbamate; 1540 s (amide II); 3065 w, 3040 w, 3019 w, 1451 m, 758 m, 739 s (ring). HRMS (ESI) calc for C_19_H_18_O_4_N_4_Na [M + Na]^+^ 389.12203, found: 389.12226.

Experimental procedures and analytical data for compound **30** are provided in the supporting information.

### 2‐(*S*)‐(*tert*‐Butoxycarbonylamino)‐3‐aminopropionic Acid **31**

Intermediate **30** (18.9 g; 81.4 mmol) was suspended in a mixture of acetonitrile (90 ml), 90 ml ethyl acetate (90 ml) and water (45 ml), and PIDA (31.4 g; 97.7 mmol) was added in five portions during 15 min. Then, 10 min after the addition of all amount of PIDA, the slurry turned clear followed by a rapid precipitation of the crude product. The cake of the filtrate was washed out with 200 ml of chilled ethyl acetate, and no additional purification was needed. Yield 12.7 g (77%). White solid, m.p. 209–211 °C. *R*_f_ = 0.40 (ethyl acetate–acetone–methanol–water 4 : 1 : 1 : 1). [*α*] 20D = −5.3 (*c* = 0.318; acetic acid). ^1^H NMR (600 MHz, DMSO): 1.39 (9H, s, (CH_3_)_3_), 2.77 (1H, dd, *J* = 11.9 and 9.1, –C**Ha**Hb–N), 3.01 (1H, dd, *J* = 11.9 and 5.4, –CHa**Hb**–N), 3.67 (1H, ddd, *J* = 9.1, 6.0 and 5.4, >CH–Ν), 6.28 (1H, br d, *J* = 6.0, –NH–CO). ^13^C NMR (150.9 MHz, DMSO): 28.33 ((CH_3_)_3_), 40.65 (–CH_2_–N), 51.08 (>CH–Ν), 78.42 (O–**C**(CH_3_)_3_), 155.35 (N–CO–Ο), 171.18 (–COOH). IR (KBr) *ν*_max_ (cm^−1^) 3348 m (NH); 1714 vs (C=O) acid; 1684 vs (C=O) carbamate; 1530 m (amide II); 1624 s (NH_2_); 2978 m, 2932 m, 1366 m (CH_3_). HRMS (ESI) calc for C_18_H_15_O_4_N_2_ [M − H]^+^ 203.10373, found: 203.10366.

### 2‐(*S*)‐(*tert‐*Butoxycarbonylamino)‐3‐azidopropionic Acid **32**

With the previously described azido transfer reaction employed for **11**, the reaction of **31** (12.7 g; 62.2 mmol), TEA (18.9 g; 186.6 mmol) and CuSO_4_·5H_2_O (155 mg; 0.622 mmol) in a mixture of 100 ml of methanol and 50 ml of water with TfN_3_ in dichloromethane gave **32**. TfN_3_ was prepared by the reaction of NaN_3_ (40.4 g; 622 mmol) and triflic anhydride (35.1 g; 124.4 mmol) in 100 ml of water and 100 ml of DCM. A bright yellow oil (9 g) was obtained after the isolation by flash chromatography on silica gel and was used as a crude product in the following step.

### Copper(II) Complex of *N* ^*δ*^‐*tert*‐Butoxycarbonyl‐l‐ornithine **35**

Complex **35** was prepared according to the literature 58 by a method starting from l‐ornithine. HCl **33** (8.4 g; 50 mmol), Cu(CH_3_COO)_2_·H_2_O (5 g; 25 mmol), Boc_2_O (12 g; 55 mmol) and 50 ml of 2 m NaOH. Yield 11.8 g (90%). Dark violet solid, >220 °C (decay). Because of the diamagnetism of copper, NMR spectra were not recorded. IR (KBr) *ν*_max_ (cm^−1^) 1684 vs (C=O) carbamate; 1573 m, 1401 s (COO^−^); 1620 vs (NH_2_); 1522 s (amide II); 2979 m, 1392 s, 1367 (CH_3_); 1174 vs (C(CH_3_)_3_). HRMS (ESI) calc for C_20_H_39_O_8_N_4_Cu [M + 1]^+^ 526.20584, found: 526.20605.

### Copper(II) Complex of *N* ^*ω*^‐*tert*‐Butoxycarbonyl‐l‐lysine **36**

With the method employed for **35**, the complex of **36** was prepared from l‐lysine·HCl **26** (9.13 g; 50 mmol), Cu(CH_3_COO)_2_·H_2_O (5 g; 25 mmol), Boc_2_O (12 g; 55 mmol) and 50 ml of 2 M NaOH. Yield 12.7 g (92%). Dark violet solid, >200 °C (decay). Because of the diamagnetism of copper, NMR spectra were not recorded. IR (KBr) *ν*_max_ (cm^−1^) 1686 vs (C=O) carbamate; 1573 m, 1401 s (COO^−^); 1623 vs (NH_2_); 1522 m (amide II); 2979 m, 1392 s, 1367 (CH_3_); 2934 m, 1456 m (CH_2_); 1173 vs (C(CH_3_)_3_). HRMS (ESI) calc for C_22_H_43_O_8_N_4_Cu [M + 1]^+^ 554.23714, found: 554.23719.

### 5‐(*tert‐*Butoxycarbonylamino)‐2‐(*S*)‐aminopentanoic Acid **37**

Protected acid **37** was prepared by the reaction of **35** (10.2 g; 19.4 mmol) and 8‐quinolinol (7.3 g; 50.4 mmol), using the method described previously 56, 57, 58, 59. Yield 8.1 g (90%). White solid, m.p. 219–222 °C. *R*_f_ = 0.63 (isopropyl alcohol‐concentrated aqueous ammonia‐water 7 : 1 : 2). [*α*] 20D = +16.7 (*c* = 0.222; glacial acetic acid). ^1^H NMR (500 MHz, DMSO): 1.37 (9H, s, (CH_3_)_3_), 1.42 (2H, m, –CH_2_–), 1.51 and 1.67 (2H, 2× m, –CH_2_–), 2.88 (2H, m, –CH_2_–N), 3.07 (1H, br t, *J* = 6.0, >CH‐Ν), 6.86 (1H, br t, *J* = 5.4, –NH–CO). ^13^C NMR (125.7 MHz, DMSO): 26.14 (–CH_2_–), 28.48 ((CH_3_)_3_), 28.80 (–CH_2_–), 54.26 (>CH–Ν), 77.52 (O–**C**(CH_3_)_3_), 155.75 (N–CO–Ο), 169.75 (–COOH). IR (KBr) *ν*_max_ (cm^−1^) 3362 s (NH); 2978 vs, 1393 s, 1366 s (CH_3_); 1174 vs (C(CH_3_)_3_); 1688 vs (C=O) carbamate; 1527 s (amide II); ~1588 vs, 1407 vs (COO^−^); 1588 s (NH +3). HRMS (ESI) calc for C_10_H_21_O_4_N_2_ [M + 1]^+^ 233.14958, found: 233.14959.

### 6‐(*tert‐*Butoxycarbonylamino)‐2‐(*S*)‐aminohexanoic Acid **38**

With the use of the same method as for **37**, protected acid **38** was prepared by the reaction of **36** (12.3 g; 22 mmol) and 8‐quinolinol (8.3 g; 57.2 mmol). Yield 9.9 g (88%). White solid, m.p. 228–230 °C. *R*_f_ = 0.64 (isopropyl alcohol–concentrated aqueous ammonia–water 7 : 1 : 2). [α] 20D = +4.8 (*c* = 0.270; glacial acetic acid). ^1^H NMR (600 MHz, DMSO): 1.28 (2H, m, –CH_2_–), 1.34 (2H, m, –CH_2_–), 1.37 (9H, s, (CH_3_)_3_), 1.53 and 1.68 (2H, 2× m, –CH_2_–), 2.88 (2H, m, –CH_2_–N), 3.06 (1H, dd, *J* = 7.3 and 5.0, >CH–Ν), 6.74 (1H, br t, *J* = 5.5, –NH–CO). ^13^C NMR (150.9 MHz, DMSO): 22.72 (–CH_2_–), 28.45 ((CH_3_)_3_), 29.41 (–CH_2_–), 31.04 (–CH_2_–), 39.95 (–CH_2_–N), 54.34 (>CH–Ν), 77.51 (O–**C**(CH_3_)_3_), 155.70 (N–CO–Ο), 169.78 (−COOH). IR (KBr) *ν*_max_ (cm^−1^) 3380 s (NH); 2978 vs, 1398 s, 1366 s (CH_3_); 1178 vs (C(CH_3_)_3_); 1689 vs (C=O) carbamate; 1520 s (amide II); 1624 vs (NH_2_); 1585 vs, 1407 vs (COO^−^); 1588 s (NH +3). HRMS (ESI) calc for C_11_H_22_O_4_N_2_Na [M + Na]^+^ 269.14718, found: 269.14719.

### 5‐(*tert‐*Butoxycarbonylamino)‐2‐(*S*)‐(9‐fluorenylmethyloxycarbonylamino)pentanoic Acid **39**

Compound **37** (5.9 g; 25.4 mmol) was placed in a 1 l round‐bottom flask, equipped with a magnetic spin bar and dissolved in a solution of NaHCO_3_ (4.3 g; 50.8 mmol) in 100 ml of water. The flask was immersed in an ice cooling bath and Fmoc‐OSu (8.6 g; 25.4 mmol) in 100 ml of dioxane was added dropwise under vigorous stirring during 30 min. When the addition of Fmoc‐OSu was complete, the reaction mixture was allowed to react for 1 h at 0 °C and then overnight at RT. Thereafter, 200 ml of water was added and followed by the dropwise addition of concentrated citric acid until pH ~ 2–3 was reached. The reaction mixture was extracted four times with 150 ml of ethyl acetate. The combined organic layers were washed twice with 150 ml of brine and twice with 150 ml of water and dried over Na_2_SO_4_. The filtrate was evaporated and the resulting brown oil was subjected to flash chromatography on silica gel, using a linear gradient ethyl acetate in ethyl acetate‐acetone‐methanol‐water 6 : 1 : 1 : 0.5. The yellow oil was triturated in a mixture of ethyl acetate–petroleum ether at −20 °C to afford the pure product. Yield 10.7 g (93%). White solid, m.p. 88–90 °C. *R*_f_ = 0.69 (ethyl acetate–acetone–methanol–water 6 : 1 : 1 : 0.5). [*α*] 20D = −1.6 (*c* = 0.254; DMF). ^1^H NMR (500 MHz, DMSO): 1.37 (9H, s, (CH_3_)_3_), 1.43 (2H, m, −CH_2_–), 1.56 and 1.71 (2H, 2× m, −CH_2_–), 2.91 (2H, m, −CH_2_–N), 3.90 (1H, ddd, *J* = 9.1, 8.2 and 4.8, >CH–Ν), 4.22 (1H, dd, *J* = 7.1 and 6.8, >CH–), 4.26 (1H, dd, *J* = 10.0 and 6.3, CO–O–C**Ha**Hb–), 4.28 (1H, dd, *J* = 10.0 and 7.1, CO–O–CHa**Hb**–), 6.80 (1H, t, *J* = 5.6, –NH–CO–O), 7.52 (1H, br d, *J* = 8.2, –NH–CO–O), 7.33 (2H, m, Ar–H), 7.41 (2H, m, Ar–H), 7.72 (2H, m, Ar–H), 7.89 (2H, m, Ar–H), 12.70 (1H, br s, COOH). ^13^C NMR (125.7 MHz, DMSO): 26.46 (–CH_2_–), 28.47 ((CH_3_)_3_), 28.62 (–CH_2_–), 40.00 (–CH_2_–N), 54.13 (>CH–Ν), 65.78 (–CH_2_–O–CO), 77.58 (O–**C**(CH_3_)_3_), 120.30(2), 125.50, 125.51, 127.28(2) and 127.83(2) (8× Ar=CH–), 140.91, 140.92, 144.02 and 144.08 (4× Ar>C=), 155.79 (N–CO–Ο), 156.24 (N–CO–Ο), 174.30 (–COOH). IR (KBr) *ν*_max_ (cm^−1^) 3348 s (NH); 2976 vs, 1393 s, 1366 s (CH_3_); 1169 vs (C(CH_3_)_3_); 1716 vs (C=O) acid; 1698 vs (C=O) carbamates; 1528 s (amide II); 3066 w, 3041 w, 1451 m, 1105 m, 759 m, 740 m (ring). HRMS (ESI) calc for C_25_H_30_O_6_N_2_Na [M + Na]^+^ 477.19961, found: 477.19964.

### 6‐(*tert‐*Butoxycarbonylamino)‐2‐(*S*)‐(9‐fluorenylmethyloxycarbonylamino)hexanoic Acid **40**

Acid **40** was prepared by the reaction of **38** (9.3 g; 35.2 mmol), NaHCO_3_ (5.9 g; 70.4 mmol) and Fmoc‐OSu (11.1 g; 35.2 mmol), using the protocol described for **39**. Yield 15.6 g (95%). White solid, m.p. 125–127 °C. *R*_f_ = 0.75 (ethyl acetate–acetone–methanol–water 6 : 1 : 1 : 0.5). [*α*] 20D = −8.5 (*c* = 0.272; DMF). ^1^H NMR (500 MHz, DMSO): 1.30 (2H, m, –CH_2_–), 1.36 (9H, s, (CH_3_)_3_), 1.36 (2H, m, –CH_2_–), 1.59 and 1.69 (2H, 2× m, –CH_2_–), 2.90 (2H, m, –CH_2_–N), 3.89 (1H, ddd, *J* = 9.2, 8.2 and 4.5, >CH–Ν), 4.22 (1H, m, >CH–), 4.27 (2H, m, CO–O–CH_2_–), 6.79 (1H, br t, *J* = 5.6, –NH–CO–O), 7.54 (1H, br d, *J* = 8.2, –NH–CO–O), 7.33 (2H, m, Ar–H), 7.41 (2H, m, Ar–H), 7.73 (2H, m, Ar–H), 7.89 (2H, m, Ar–H). ^13^C NMR (125.7 MHz, DMSO): 23.17 (–CH_2_–), 28.49 ((CH_3_)_3_), 29.34 (–CH_2_–), 30.83 (–CH_2_–), 39.82 (–CH_2_–N), 46.88 (>CH–), 54.21 (>CH–Ν), 65.78 (–CH_2_–O–CO), 77.56 (O–**C**(CH_3_)_3_), 120.33, 120.34, 125.50, 125.52, 127.29(2) and 127.85(2) (8× Ar=CH–), 140.93, 140.94, 144.03 and 144.09 (4× Ar>C=), 155.80 (N–CO–Ο), 156.32 (N–CO–Ο), 174.40 (–COOH). IR (KBr) *ν*_max_ (cm^−1^) 3391 s, 3369 s (NH); 2978 vs, 1393 s, 1367 s (CH_3_); 2936 m, 1478 m (CH_2_); 1173 vs (C(CH_3_)_3_); 1711 vs (C=O) acid; 1693 vs + br (C=O) carbamates; 1525 s (amide II); 3067 w, 3041 w, 1451 m, 1105 m, 760 m, 740 m (ring). HRMS (ESI) calc for C_26_H_32_O_6_N_2_Na [M + Na]^+^ 491.21526, found: 491.21527.

### 5‐Azido‐2‐(*S*)‐(9‐fluorenylmethyloxycarbonylamino)pentanoic Acid **41**

Compound **39** (10.4 g; 22.9 mmol) was treated with 20 ml of DCM, 20 ml of TFA and 2 ml of water. After 2 h stirring, volatile materials were evaporated to give a yellow oil, which was then suspended with NaHCO_3_ (19.2 g; 229 mmol) and CuSO_4_·5H_2_O (57 mg; 0.229 mmol) in 100 ml of water and 150 ml of methanol. TfN_3_, prepared by the reaction of NaN_3_ (14.9 g; 229 mmol) and triflic anhydride (12.9 g; 45.8 mmol), was added dropwise to the slurry in 100 ml of DCM, followed by the work‐up, which was the same as in the case of the diazotransfer reaction leading to the product **14**. Yield 8 g (92%). White solid, 127–128 °C. *R*_f_ = 0.71 (ethyl acetate–acetone–methanol–water 6 : 1 : 1 : 0.5). [*α*] 20D = −6.7 (*c* = 0.254; DMF). ^1^H NMR (500 MHz, DMSO): 1.59 (2H, m, –CH_2_–), 1.66 and 1.78 (2H, 2× m, –CH_2_–), 3.34 (2H, m, –CH_2_–N_3_), 3.98 (1H, ddd, *J* = 9.2, 8.2 and 5.0, >CH–Ν), 4.23 (1H, br t, *J* = 7.0, >CH–), 4.30 (2H, m, CO–O–CH_2_–), 7.33 (2H, m, Ar–H), 7.42 (2H, m, Ar–H), 7.70 (1H, br d, *J* = 8.2, –NH–CO–O), 7.73 (2H, m, Ar–H), 7.89 (2H, m, Ar–H), 12.66 (1H, br s, COOH). ^13^C NMR (125.7 MHz, DMSO): 25.31 (–CH_2_–), 28.21 (–CH_2_–), 46.89 (>CH–), 50.45 (–CH_2_–N_3_), 53.57 (>CH–Ν), 65.81 (–CH_2_–O–CO), 120.33, 120.35, 125.48, 125.50, 127.28(2) and 127.86(2) (8× Ar=CH–), 140.94, 140.96, 144.00 and 144.07 (4× Ar>C=), 156.38 (N–CO–Ο), 173.88 (–COOH). IR (KBr) *ν*_max_ (cm^−1^) 3391 s, 3339 s (NH); 2874 m (CH_2_); 2096 vs (N_3_); 1711 vs, (C=O) acid; 1729 vs, 1687 vs (C=O) carbamate; 1534 s (amide II); 3043 m, 1451 s, 1103 m, 1032 m, 759 s, 737 s, 622 s (ring). HRMS (ESI) calc for C_20_H_20_O_4_N_4_Na [M + Na]^+^ 403.13768, found: 403.13774.

### 6‐Azido‐2‐(*S*)‐(9‐fluorenylmethyloxycarbonylamino)hexanoic Acid **42**

With the procedure described for **41**, acid **42** was prepared starting from **40** (14.9 g; 31.8 mmol), NaHCO_3_ (26.7 g; 318 mmol), CuSO_4_·5H_2_O (79 mg; 0.32 mmol), NaN_3_ (20.7 g; 318 mmol) and triflic anhydride (17.9 g; 63.6 mmol). Yield 11.1 g (89%). White solid, 73–75 °C. *R*_f_ = 0.74 (ethyl acetate–acetone–methanol–water 6 : 1 : 1 : 0.5). [*α*] 20D = −15.7 (*c* = 0.261; DMF). ^1^H NMR (500 MHz, DMSO): 1.38 (2H, m, –CH_2_–), 1.53 (2H, m, –CH_2_–), 1.63 and 1.73 (2H, 2× m, –CH_2_–), 3.32 (2H, t, *J* = 6.8, –CH_2_–N_3_), 3.94 (1H, ddd, *J* = 9.5, 8.0 and 4.6, >CH–Ν), 4.22 (1H, br t, *J* = 7.0, >CH–), 4.28 (2H, m, CO–O–CH_2_–), 7.33 (2H, m, Ar–H), 7.42 (2H, m, Ar–H), 7.67 (1H, br d, *J* = 8.0, –NH–CO–O), 7.73 (2H, m, Ar–H), 7.89 (2H, m, Ar–H), 12.62 (1H, br s, COOH). ^13^C NMR (125.7 MHz, DMSO): 23.14 (–CH_2_–), 28.06 (–CH_2_–), 30.50 (–CH_2_–), 46.87 (>CH–), 50.73 (–CH_2_–N_3_), 53.88 (>CH–Ν), 65.81 (–CH_2_–O–CO), 120.34, 120.35, 125.49, 125.52, 127.28(2) and 127.87(2) (8× Ar=CH–), 140.94, 140.95, 144.02 and 144.07 (4× Ar>C=), 156.40 (N–CO–Ο), 174.11 (–COOH). IR (KBr) *ν*_max_ (cm^−1^) 3378 m (NH); 2937 m, 2886 m, 1477 m (CH_2_); 2097 vs (N_3_); 1717 vs, (C=O) acid; 1746 vs, 1698 vs (C=O) carbamate; 1525 s (amide II); 3065 m, 3043 m, 1451 s, 1102 m, 758 s, 739 s (ring). HRMS (ESI) calc for C_21_H_22_O_4_N_4_Na [M + Na]^+^ 417.15333, found: 417.15346.

### General Protocol for the Manual Synthesis of Tripeptides **43**–**47**

- Rink amide resin (400 μmol, loading 0.68 mmol/g) was placed in a 20 ml polypropylene syringe equipped with a polypropylene frit and swelled in 10 ml of DMF for 1 h.
- Fmoc group was cleaved by treatment with 20% piperidine/DMF (5 ml for 5 and 20 min), followed by five washings with 5 ml of DMF.
- Fmoc‐Phe (619 mg; 1.6 mmol), HBTU (607 mg; 1.6 mmol) and DIPEA (418 μmol; 2.4 mmol) were added in 5 ml of DMF. The resin was stirred for 2 h and then washed five times with 5 ml of DMF. Step (iii) was repeated, and the resin was washed successively with DMF, MeOH, DCM and DMF (each solvent five times with 5 ml). Step (ii) was repeated. Step (iii) was repeated twice (Fmoc‐Val was used in the case of tripeptide **43**; 543 mg; 1.6 mmol). Step (ii) was repeated.
- **14** (563 mg; 1.6 mmol) or **29** (586 mg; 1.6 mmol) or **41** (608 mg; 1.6 mmol) or **42** (630 mg; 1.6 mmol) with HBTU (607 mg; 1.6 mmol) and DIPEA (418 μmol; 2.4 mmol) in 5 ml of DMF was added, stirred for 2 h and then washed five times with 5 ml of DMF. Only in the case of the synthesis of **44** did couplings with **14** proceed for 5 and 18 h. Step (iv) was repeated and the resin successively washed with DMF, MeOH, DCM and DMF (each solvent five times with 5 ml). Step (ii) was repeated.
- Thereafter, 300 μl of Ac_2_O and 300 μl of DIPEA, each in 1 ml of DMF, were added; the resin was stirred for 15 min and washed five times with 5 ml of DMF. Step (v) was repeated. Thereafter, the resin was transported to a small glass reactor equipped with a frit, rinsed with 50 ml of dichloromethane and dried overnight under deep vacuum. The resin was cleaved for 60 min with 5 ml of a cocktail of TFA/water/triisopropylsilane (CAS 6485‐79‐6) (95/2.5/2.5). The cleavage step was repeated under the same conditions, the resin was washed with 10 ml of glacial acetic acid and all cleavage solutions and acetic acid were combined and evaporated under reduced pressure. The brown residue was then sonicated for 10 min with 10 ml diethyl ether in an ice cooling bath. The slurry was centrifuged for 10 min at 10 000 *g*, diethyl ether was decanted and crude amorphous white tripeptides were dried *in vacuo*. The purity of the prepared tripeptides was checked by RP‐HPLC; analytical samples were isolated using the following gradient: *t* = 0 min (20% B), *t* = 30 min (100% B).

Analytical data for compounds **43**–**47** are provided in the supporting information.

### CH_3_COCO‐Phe‐Phe‐CONH_2_ **48**

Yield 49 mg (32%). Lyophilisate. [*α*] 20D = −14.3 (*c* = 0.119; DMSO). ^1^H NMR (500 MHz, DMSO): 2.25 (3H, s, CH_3_–CO), 2.81 (1H, dd, *J* = 13.8 and 9.1, –C**Ha**Hb–), 3.02 (1H, dd, *J* = 13.8 and 5.0, –CHa**Hb**–), 2.89 (1H, dd, *J* = 13.8 and 9.3, –C**Ha**Hb–), 2.96 (1H, dd, *J* = 13.8 and 4.7, –CHa**Hb**–), 4.44 (1H, ddd, *J* = 9.3, 8.7 and 4.7, >CH–N), 4.45 (1H, ddd, *J* = 9.1, 8.2 and 5.0, >CH–N), 7.12 and 7.42 (2H, 2× br s, CONH_2_), 7.12–7.26 (10H, m, 2× C_6_H_5_), 8.21 (1H, d, *J* = 8.2, –NH–CO), 8.37 (1H, d, *J* = 8.7, –NH–CO). ^13^C NMR (125.7 MHz, DMSO): 24.98 (**C**H_3_–CO), 37.23 (–CH_2_–), 37.92 (–CH_2_–), 54.02 (>CH–N), 54.36 (>CH–N), 126.52, 126.57, 128.28(4), 129.40(2) and 129.46(2) (10× Ar=CH–), 137.62 and 137.98 (2× Ar>C=), 160.61 (–CO–**C**O–N), 169.99 (N–CO–), 172.86 (–CONH_2_), 196.73 (–**C**O–CO–N). IR (KBr) *ν*_max_ (cm^−1^) 3408 vs, 3320 (NH); 1725 m (C=O) ketone, 1665 s, 1640 vs (C=O) amides; 1524 m (amide II); 3086 w, 3065 w, 3029 w, 1498 w, 1455 w, 750 w, 703 w (ring); 1358 m (CH_3_). HRMS (ESI) calc for C_21_H_23_O_4_N_3_Na [M + Na]^+^ 404.15808 found: 404.15815.

## Supporting information

Supporting info item

## References

[1] Tornoe CW , Christensen C , Meldal M . Peptidotriazoles on solid phase: 1,2,3‐triazoles by regiospecific copper(I)‐catalyzed 1,3‐dipolar cycloadditions of terminal alkynes to azides. J. Org. Chem. 2002; 67: 3057–3064. 11975567 10.1021/jo011148j

[2] Rostovtsev VV , Green LG , Fokin VV , Sharpless KB . A stepwise Huisgen cycloaddition process: copper(I)‐catalyzed regioselective ‘ligation’ of azides and terminal alkynes. Angew. Chem.‐Int. Edit. 2002; 41: 2596–2599. 10.1002/1521-3773(20020715)41:14<2596::AID-ANIE2596>3.0.CO;2-4 12203546

[3] Himo F , Lovell T , Hilgraf R , Rostovtsev VV , Noodleman L , Sharpless KB , Fokin VV . Copper(I)‐catalyzed synthesis of azoles. DFT study predicts unprecedented reactivity and intermediates. J. Am. Chem. Soc. 2005; 127: 210–216. 15631470 10.1021/ja0471525

[4] Bock VD , Hiemstra H , van Maarseveen JH . Cu‐I‐catalyzed alkyne‐azide ‘click’ cycloadditions from a mechanistic and synthetic perspective. Eur. J. Org. Chem. 2006; 51–68.

[5] Ingale S , Dawson PE . On resin side‐chain cyclization of complex peptides using CuAAC. Org. Lett. 2011; 13: 2822–2825. 21553819 10.1021/ol200775h

[6] Jagasia R , Holub JM , Bollinger M , Kirshenbaum K , Finn MG . Peptide cyclization and cyclodimerization by Cu‐I‐mediated azide‐alkyne cycloaddition. J. Org. Chem. 2009; 74: 2964–2974. 19309103 10.1021/jo802097m PMC2677176

[7] Pedersen DS , Abell A . 1,2,3‐Triazoles in peptidomimetic chemistry. Eur. J. Org. Chem. 2011; 2399–2411.

[8] Angell YL , Burgess K . Peptidomimetics via copper‐catalyzed azide‐alkyne cycloadditions. Chem. Soc. Rev. 2007; 36: 1674–1689. 17721589 10.1039/b701444a

[9] Park JH , Waters ML . Positional effects of click cyclization on beta‐hairpin structure, stability, and function. Org. Biomol. Chem. 2013; 11: 69–77. 23064223 10.1039/c2ob26445e PMC4037565

[10] Kawamoto SA , Coleska A , Ran X , Yi H , Yang CY , Wang SM . Design of triazole‐stapled BCL9 alpha‐helical peptides to target the beta‐catenin/B‐cell CLL/lymphoma 9 (BCL9) protein–protein interaction. J. Med. Chem. 2012; 55: 1137–1146. 22196480 10.1021/jm201125d PMC3286869

[11] Scrima M , Le Chevalier‐Isaad A , Rovero P , Papini AM , Chorev M , D'Ursi AM . Cu‐I‐catalyzed azide‐alkyne intramolecular *i*‐to‐(*i* + 4) side‐chain‐to‐side‐chain cyclization promotes the formation of helix‐like secondary structures. Eur. J. Org. Chem. 2010; 446–457.

[12] Holland‐Nell K , Meldal M . Maintaining biological activity by using triazoles as disulfide bond mimetics. Angew. Chem.‐Int. Edit. 2011; 50: 5204–5206. 10.1002/anie.201005846 21472909

[13] Meldal M , Tornoe CW . Cu‐catalyzed azide‐alkyne cycloaddition. Chem. Rev. 2008; 108: 2952–3015. 18698735 10.1021/cr0783479

[14] Holub JM , Kirshenbaum K . Tricks with clicks: modification of peptidomimetic oligomers via copper‐catalyzed azide‐alkyne [3 + 2] cycloaddition. Chem. Soc. Rev. 2010; 39: 1325–1337. 20309489 10.1039/b901977b

[15] Castro V , Rodriguez H , Abericio F . CuAAC: an efficient click chemistry reaction on solid phase. ACS Comb. Sci. 2016; 18: 1–14. 26652044 10.1021/acscombsci.5b00087

[16] Nilsson BL , Kiessling LL , Raines RT . Staudinger ligation: a peptide from a thioester and azide. Org. Lett. 2000; 2: 1939–1941. 10891196 10.1021/ol0060174

[17] Vikova J , Collinsova M , Kletvikova E , Budesinsky M , Kaplan V , Zakova L , Veverka V , Hexnerova R , Avino RJT , Strakova J , Selicharova I , Vanek V , Wright DW , Watson CJ , Turkenburg JP , Brzozowski AM , Jiracek J . Rational steering of insulin binding specificity by intra‐chain chemical crosslinking. Sci. Rep. 2016; 6: 12. 26792393 10.1038/srep19431 PMC4726324

[18] Wei L , Lubell WD . Scope and limitations in the use of N‐(PhF)serine‐derived cyclic sulfamidates for amino acid synthesis. Can. J. Chem. 2001; 79: 94–104.

[19] Sai Sudhir V , Phani Kumar NY , Nasir Baig RB , Chandrasekaran S . Facile entry into triazole fused heterocycles via sulfamidate derived azido‐alkynes. J. Org. Chem 2009; 74: 7588–7591. 19725506 10.1021/jo9016748

[20] Sudhir VS , Kumar NYP , Chandrasekaran S . Click chemistry inspired synthesis of ferrocene amino acids and other derivatives. Tetrahedron 2010; 66: 1327–1334.

[21] Arnold LD , May RG , Vederas JC . Synthesis of optically pure alpha‐amino‐acids via salts of alpha‐amino‐beta‐propiolactone. J. Am. Chem. Soc. 1988; 110: 2237–2241.

[22] Fujii M , Hidaka J . Nucleic acid analog peptide containing beta‐aminoalanine modified with nucleobases. Nucleos. Nucleot. 1999; 18: 1421–1422.

[23] Sun DQ , Jones V , Carson EI , Lee REB , Scherman MS , McNeil MR , Lee RE . Solid‐phase synthesis and biological evaluation of a uridinyl branched peptide urea library. Bioorg. Med. Chem. Lett. 2007; 17: 6899–6904. 17962016 10.1016/j.bmcl.2007.09.097 PMC2140236

[24] Fujii M , Yoshida K , Hidaka J , Ohtsu T . Nucleic acid analog peptide (NAAP) .2. Syntheses and properties of novel DNA analog peptides containing nucleobase linked beta‐aminoalanine. Bioorg. Med. Chem. Lett. 1997; 7: 637–640.

[25] Fujii M , Yoshida K , Hidaka J , Ohtsu T . Hybridization properties of nucleic acid analogs containing beta‐aminoalanine modified with nucleobases. Chem. Commun. 1998; 717–718.

[26] Rosenberg SH , Spina KP , Woods KW , Polakowski J , Martin DL , Yao ZL , Stein HH , Cohen J , Barlow JL , Egan DA , Tricarico KA , Baker WR , Kleinert HD . Studies directed toward the design of orally active renin inhibitors. 1. Some factors influencing the absorption of small peptides. J. Med. Chem. 1993; 36: 449–459. 8474101 10.1021/jm00056a005

[27] Kogan TP , Rawson TE . The synthesis of chiral 3‐oxo‐6‐(phenylmethoxy)‐carbonyl‐2‐piperazineacetic acid‐esters designed for the presentation of an aspartic‐acid side‐chain – a subsequent novel Friedel crafts reaction. Tetrahedron Lett. 1992; 33: 7089–7092.

[28] Pickersgill IF , Rapoport H . Preparation of functionalized, conformationally constrained DTPA analogues from l‐ or d‐serine and trans‐4‐hydroxy‐l‐proline. Hydroxymethyl substituents on the central acetic acid and on the backbone. J. Org. Chem. 2000; 65: 4048–4057. 10866623 10.1021/jo000071g

[29] Gajewski M , Seaver B , Esslinger CS . Design, synthesis, and biological activity of novel triazole amino acids used to probe binding interactions between ligand and neutral amino acid transport protein SN1. Bioorg. Med. Chem. Lett. 2007; 17: 4163–4166. 17561393 10.1016/j.bmcl.2007.05.061 PMC2045077

[30] Colombo R , Mingozzi M , Belvisi L , Arosio D , Piarulli U , Carenini N , Perego P , Zaffaroni N , De Cesare M , Castiglioni V , Scanziani E , Gennari C . Synthesis and biological evaluation (*in vitro* and *in vivo*) of cyclic arginine–glycine–aspartate (RGD) peptidomimetic‐paclitaxel conjugates targeting integrin alpha(v)beta(3). J. Med. Chem. 2012; 55: 10460–10474. 23140358 10.1021/jm301058f

[31] Otsuka M , Kittaka A , Iimori T , Yamashita H , Kobayashi S , Ohno M . Synthetic studies on an antitumor antibiotic, bleomycin. 12. Preparation of an l‐2,3‐diaminopropionic acid‐synthetic intermediate. Chem. Pharm. Bull. 1985; 33: 509–514. 2410151 10.1248/cpb.33.509

[32] Stanley NJ , Pedersen DS , Nielsen B , Kvist T , Mathiesen JM , Brauner‐Osborne H , Taylor DK , Abell AD . 1,2,3‐Triazolyl amino acids as AMPA receptor ligands. Bioorg. Med. Chem. Lett. 2010; 20: 7512–7515. 21036612 10.1016/j.bmcl.2010.09.139

[33] Boger DL , Honda T , Menezes RF , Colletti SL , Dang Q , Yang WJ . Total syntheses of (+)‐P‐3A, epi‐(−)‐P‐3A, and (−)‐desacetamido P‐3A. J. Am. Chem. Soc. 1994; 116: 82–92.

[34] Mukai S , Flematti GR , Byrne LT , Besant PG , Attwood PV , Piggott MJ . Stable triazolylphosphonate analogues of phosphohistidine. Amino Acids 2012; 43: 857–874. 22105612 10.1007/s00726-011-1145-2

[35] Panda G , Rao NV . A short synthetic approach to chiral serine azido derivatives. Synlett 2004; 714–716. DOI: 10.1055/s-2004-817770.

[36] Jeong JM , Shetty D , Lee DS , Chung JK , Lee MC , Jeong J , Dineswi S , Lee D , US2012029177‐A1, 2011.

[37] Friscourt F , Fahrni CJ , Boons GJ . A fluorogenic probe for the catalyst‐free detection of azide‐tagged molecules. J. Am. Chem. Soc. 2012; 134: 18809–18815. 23095037 10.1021/ja309000s PMC3525324

[38] Zhong M , Hanan EJ , Shen W , Bui M , Arkin MR , Barr KJ , Evanchik MJ , Hoch U , Hyde J , Martell JR , Oslob JD , Paulvannan K , Prabhu S , Silverman JA , Wright J , Yu CH , Zhu JA , Flanagan WM . Structure–activity relationship (SAR) of the alpha‐amino acid residue of potent tetrahydroisoquinoline (THIQ)‐derived LFA‐1/ICAM‐1 antagonists. Bioorg. Med. Chem. Lett. 2011; 21: 307–310. 21109434 10.1016/j.bmcl.2010.11.014

[39] Zou Y , Fahmi NE , Vialas C , Miller GM , Hecht SM . Total synthesis of deamido bleomycin A(2), the major catabolite of the antitumor agent bleomycin. J. Am. Chem. Soc. 2002; 124: 9476–9488. 12167044 10.1021/ja012741l

[40] Zhang XJ , Krishnamurthy R . Mapping the landscape of potentially primordial informational oligomers: oligo‐dipeptides tagged with orotic acid derivatives as recognition elements. Angew. Chem.‐Int. Edit. 2009; 48: 8124–8128. 10.1002/anie.200904188 19768828

[41] Lau YH , Spring DR . Efficient synthesis of Fmoc‐protected azido amino acids. Synlett 2011; 1917–1919. DOI: 10.1055/s-0030-1260950.

[42] Akaji K , Aimoto S . Synthesis of MEN11420, a glycosylated bicyclic peptide, by intramolecular double cyclization using a chloroimidazolinium coupling reagent. Tetrahedron 2001; 57: 1749–1755.

[43] Hirschmann R , Yao WQ , Arison B , Maechler L , Rosegay A , Sprengeler PA , Smith AB . Synthesis of the first tricyclic homodetic peptide. Use of coordinated orthogonal deprotection to achieve directed ring closure. Tetrahedron 1998; 54: 7179–7202.

[44] Barghash RF , Massi A , Dondoni A . Synthesis of thiourea‐tethered C‐glycosyl amino acids via isothiocyanate‐amine coupling. Org. Biomol. Chem. 2009; 7: 3319–3330. 19641791 10.1039/b908156a

[45] Zhang LH , Kauffman GS , Pesti JA , Yin JG . Rearrangement of *N*‐alpha‐protected l‐asparagines with iodosobenzene diacetate. A practical route to beta‐amino‐l‐alanine derivatives. J. Org. Chem. 1997; 62: 6918–6920.

[46] Millward SW , Henning RK , Kwong GA , Pitram S , Agnew HD , Deyle KM , Nag A , Hein J , Lee SS , Lim J , Pfeilsticker JA , Sharpless KB , Heath JR . Iterative *in situ* click chemistry assembles a branched capture agent and allosteric inhibitor for Akt1. J. Am. Chem. Soc. 2011; 133: 18280–18288. 21962254 10.1021/ja2064389 PMC3651860

[47] Ghosh PS , Hamilton AD . Noncovalent template‐assisted mimicry of multiloop protein surfaces: assembling discontinuous and functional domains. J. Am. Chem. Soc. 2012; 134: 13208–13211. 22839643 10.1021/ja305360q

[48] Pehere AD , Abell AD . New beta‐strand templates constrained by Huisgen cycloaddition. Org. Lett. 2012; 14: 1330–1333. 22339212 10.1021/ol3002199

[49] Oh KI , Lee JH , Joo C , Han H , Cho M . Beta‐azidoalanine as an IR probe: application to amyloid A beta(16–22) aggregation. J. Phys. Chem. B 2008; 112: 10352–10357. 18671422 10.1021/jp801558k

[50] Miller N , Williams GM , Brimble MA . Synthesis of fish antifreeze neoglycopeptides using microwave‐assisted ‘click chemistry’. Org. Lett. 2009; 11: 2409–2412. 19473046 10.1021/ol9005536

[51] Roice M , Johannsen I , Meldal M . High capacity poly(ethylene glycol) based amino polymers for peptide and organic synthesis. QSAR Comb. Sci. 2004; 23: 662–673.

[52] Thurieau C , Janiak P , Krantic S , Guyard C , Pillon A , Kucharczyk N , Vilaine JP , Fauchere JL . A new somatostatin analog with optimized ring size inhibits neointima formation induced by balloon injury in rats without altering growth‐hormone release. Eur. J. Med. Chem. 1995; 30: 115–122.

[53] Sakura N , Itoh T , Uchida Y , Ohki K , Okimura K , Chiba K , Sato Y , Sawanishi H . The contribution of the N‐terminal structure of polymyxin B peptides to antimicrobial and lipopolysaccharide binding activity. B. Chem. Soc. Jpn. 2004; 77: 1915–1924.

[54] Papeo G , Giordano P , Brasca MG , Buzzo F , Caronni D , Ciprandi F , Mongelli N , Veronesi M , Vulpetti A , Dalvit C . Polyfluorinated amino acids for sensitive F‐19 NMR‐based screening and kinetic measurements. J. Am. Chem. Soc. 2007; 129: 5665–5672. 17417847 10.1021/ja069128s

[55] Roth S , Thomas NR . A concise route to l‐azidoamino acids: l‐azidoalanine, l‐azidohomoalanine and l‐azidonorvaline. Synlett 2010; 607–609. DOI: 10.1038/nprot.2010.164.

[56] Masiukiewicz E , Wiejak S , Rzeszotarska B . Scalable syntheses of *N*‐alpha‐benzyloxycarbonyl‐l‐ornithine and of *N*‐alpha‐(9‐fluorenylmethoxy)carbonyl‐l‐ornithine. Org. Prep. Proced. Int. 2002; 34: 531–537.

[57] Wiejak S , Masiukiewicz E , Rzeszotarska B . A large scale synthesis of mono‐ and di‐urethane derivatives of lysine. Chem. Pharm. Bull. 1999; 47: 1489–1490. 10.1248/cpb.49.1189 11558610

[58] Wiejak S , Masiukiewicz E , Rzeszotarska B . Improved scalable syntheses of mono‐ and bis‐urethane derivatives of ornithine. Chem. Pharm. Bull. 2001; 49: 1189–1191. 11558610 10.1248/cpb.49.1189

[59] Bayryamov SG , Vassilev NG , Petkov DD . The two pathways for effective orthogonal protection of l‐ornithine, for amino acylation of 5′‐*O*‐pivaloyl nucleosides, describe the general and important role for the successful imitation, during the synthesis of the model substrates for the ribosomal mimic reaction. Protein Pept. Lett. 2010; 17: 889–898. 20205650 10.2174/092986610791306625

[60] Strazzolini P , Scuccato M , Giumanini AG . Deprotection of *t*‐butyl esters of amino acid derivatives by nitric acid in dichloromethane. Tetrahedron 2000; 56: 3625–3633.

[61] Chevallet P , Garrouste P , Malawska B , Martinez J . Facile synthesis of *tert*‐butyl ester of *N*‐protected amino‐acids with *tert*‐butyl bromide. Tetrahedron Lett. 1993; 34: 7409–7412.

[62] Fernandez S , Crocamo N , Incerti M , Giglio J , Scarone L , Rey A . Preparation and preliminary bioevaluation of a ^99m^Tc(CO)_3_‐glucose derivative prepared by a click chemistry route. J. Label. Compd. Radiopharm. 2012; 55: 274–280.

[63] Nishiyama K , Karigomi H . Reaction of trimethylsilyl azide with organic halides. Chem. Lett. 1982; 11: 1477–1478.

[64] Photaki I . Transformation of serine to cysteine. *β*‐Elimination reactions in serine derivatives. J. Am. Chem. Soc. 1963; 85: 1123–1126.

[65] Johansson H , Pedersen DS . Azide‐ and alkyne‐derivatised alpha‐amino acids. Eur. J. Org. Chem. 2012; 4267–4281.

[66] Photaki I , Bardakos V . Transformation of l‐serine peptides to l‐cysteine peptides. J. Am. Chem. Soc. 1965; 87: 3489–3492. 14322542 10.1021/ja01093a037

[67] Han JR , Lian JT , Tian X , Zhou SW , Zhen XL , Liu SX . Total synthesis of micromide: a marine natural product. Eur. J. Org. Chem. 2014; 7232‐7238.

[68] Inamoto A , Ogasawara K , Omata K , Kabuto K , Sasaki Y . Samarium(III)‐propylenediaminetetraacetate complex: a water‐soluble chiral shift reagent for use in high‐field NMR. Org. Lett. 2000; 2: 3543–3545. 11073640 10.1021/ol006379b

[69] Hruba L , Budesinsky M , Picha J , Jiracek J , Vanek V . Simplified syntheses of the water‐soluble chiral shift reagents Sm‐(R)‐pdta and Sm‐(S)‐pdta. Tetrahedron Lett. 2013; 54: 6296–6297.

[70] Fields GB , Noble RL . Solid‐phase peptide‐synthesis utilizing 9‐fluorenylmethoxycarbonyl amino‐acids. Int. J. Pept. Prot. Res. 1990; 35: 161–214. 10.1111/j.1399-3011.1990.tb00939.x 2191922

[71] Angelini G , Speranza M . Gas‐phase acid‐induced nucleophilic displacement‐reactions. 5. Quantitative‐evaluation of neighboring‐group participation in bifunctional compounds. J. Am. Chem.l Soc. 1981; 103: 3800–3806.

