# Supplementary material for: Optimized syntheses of Fmoc azido amino acids for the preparation of azidopeptides
Source: J Pept Sci. 2017 Jan 25;23(3):202–14. doi: 10.1002/psc.2968 (PMC5347871; doi:10.1002/psc.2968)
Supplement: Supplementary file 1 — Supporting info item [file PSC-23-202-s001.pdf]

Supporting Information

for

Optimized syntheses of Fmoc azido amino acids for the preparation of azidopeptides

Jan Pícha, Miloš Buděšínský, Kateřina Macháčková, Michaela Collinsová and Jiří Jiráček*

Institute of Organic Chemistry and Biochemistry, Czech Academy of Sciences, v.v.i., Flemingovo nám. 2, 166 10 Prague 6, Czech Republic

E-mail: jiracek@uochb.cas.cz

Table of contents:

Experimental procedures and analytical data for compound **3** …………………. Page 2

Experimental procedures and analytical data for compound **4** …………………. Page 2

Experimental procedures and analytical data for compound **5** …………………. Page 3

Experimental procedures and analytical data for compound **20** ………………… Page 4

Experimental procedures and analytical data for compound **25** ………………… Page 4

Experimental procedures and analytical data for compound **26** ………………… Page 5

Experimental procedures and analytical data for compound **30** ………………… Page 6

Analytical data for compound **43** ……………………………………………….. Page 6

Analytical data for compound **44** ……………………………………………….. Page 7

Analytical data for compound **45** ……………………………………………….. Page 7

Analytical data for compound **46** ……………………………………………….. Page 8

Analytical data for compound **47** ……………………………………………….. Page 8

2-(*S*)-(Benzyloxycarbonylamino)-3-hydroxypropanoic acid **3**

L-Serine **1** (10.5 g; 0.1 mol; $[\alpha]_D^{20}$ = +13, c = 5, 5M HCl) was dissolved in 200 ml of 10 % aqueous solution of sodium carbonate in a 1 L round-bottom flask equipped with magnetic spin bar. The flask was immersed in an ice cooling bath, and benzyl chloroformate (1.1 eq.; 18.8 g; 0.11 mol) in 100 ml of dioxane was added dropwise during a period of 30 minutes under vigorous stirring. When the addition of ZCl was completed, the reaction mixture was allowed to react for 1 h at 0 °C and then overnight at room temperature. The reaction mixture was transferred to the separatory funnel, 500 ml of water was added and the mixture extracted by 200 ml of diethyl ether. The separated aqueous-dioxane layer was poured back into the reaction flask. During cooling, conc. HCl was added dropwise under vigorously stirring until pH ≈ 1 was reached. A cloudy solution was extracted with 3 x 400 ml ethyl acetate; combined fractions were washed 2 x with 150 ml of brine and then dried with solid Na_2SO_4. The drying agent was filtered off and the filtrate evaporated under reduced pressure. Crystallization of the product from a mixture of ethyl acetate and petroleum ether afforded colorless crystals. Yield 18.3 g (77%), m.p. 114-116 °C. R_f = 0.70 (ethyl acetate-acetone-methanol-water 6 : 1: 1 : 0.5). $[\alpha]_D^{20}$ = +6.6 (c = 1.089; CH_3COOH). ^{1}H NMR (600 MHz, DMSO): 3.65 (1H, dd, *J* = 11.2 and 5.6, HO–C**<u>Ha</u>**Hb–), 3.67 (1H, dd, *J* = 11.2 and 4.5, O–CHa**<u>Hb</u>**–), 4.06 (1H, ddd, *J* = 8.2, 5.6 and 4.5, >CH–N), 5.03 (1H, d, *J* = 12.5, CO–O–C**<u>Ha</u>**Hb–), 5.05 (1H, d, *J* = 12.5, CO–O–CHa**<u>Hb</u>**–), 7.29 (1H, d, *J* = 8.2, –NH–CO), 7.31 – 7.37 (5H, m, C_6H_5); ^{13}C NMR (150.9 MHz, DMSO): 56.84 (>CH–N); 61.52 (–CH_2–O), 65.65 (–CH_2–O–CO), 127.92(2), 128.00 and 128.54(2) (5x Ar =CH–), 137.17 (Ar >C=), 156.22 (N–CO–O), 172.32 (O–CO–). IR (KBr) ν_{max} cm^{-1} 3436 s (OH); 3336 s, 3319 s (NH); 1747 vs (C=O) acid; 1690 vs (C=O) carbamate; 1534 vs (amide II); 3061 m, 3029 m, 1083 m, 1060 s, 1029 s, 697 s (ring). HRMS (ESI) calc for $C_{11}H_{12}O_5N$ $[M+H]^+$ 238.07210, found: 238.07195.

tert-Butyl 2-(*S*)-(benzyloxycarbonylamino)-3-hydroxypropanoate **4**

The solution Z-L-Ser **3** (18 g; 75.3 mmol) in 400 ml of *N*,*N*-dimethylacetamide and 200 ml of *tert*-butyl bromide was mixed with finely ground anhydrous potassium carbonate (69.1 g; 0.5 mol) and TEBAC (11.4 g; 0.05 mol). The reaction mixture was vigorously stirred and heated at 60 °C overnight. After cooling, the content of the flask was poured on to 500 g of crushed ice. The bright brown solution was extracted 4 x with 200 ml of ethyl acetate. Combined organic layers were washed 2 x with 200 ml of water and dried over sodium sulfate. The drying agent was filtered off and the filtrate was evaporated under reduced pressure to afford 22 g of crude

oily material, which was purified by flash chromatography on silica gel, using a linear gradient of ethyl acetate in toluene. The required product was a white solid and an analytical sample was prepared by crystallization from ethyl acetate and petroleum ether. Yield 14.7 g (66%), m.p. 91-92 °C. R_f = 0.69 (toluene-ethyl acetate 50 : 50). $[\alpha]_D^{20}$ = -14.6 (c = 1.006; C_2H_5OH). ^{1}H NMR (600 MHz, DMSO): 1.39 (9H, s, $(CH_3)_3$), 3.62 (1H, dt, J = 11.0, 6.6 and 6.0, HO–C**Ha**Hb–), 3.65 (1H, ddd, J = 11.0, 6.6 and 5.3, O–CHa**Hb**–), 4.00 (1H, ddd, J = 6.6, 6.0 and 4.0, >CH–N), 4.85 (1H, dd, J = 6.6 and 5.3, –OH), 5.04 (2H, s, CO–O–CH_2–), 7.34 (1H, d, J = 4.4, –NH–CO), 7.31 – 7.37 (5H, m, C_6H_5); ^{13}C NMR (150.9 MHz, DMSO): 27.85 ($(CH_3)_3$), 57.49 (>CH–N), 61.56 (–CH_2–O), 65.63 (–CH_2–O–CO), 80.77 (O–**C**$(CH_3)_3$), 127.91(2), 127.99 and 128.51(2) (5x Ar =CH–), 137.17 (Ar >C=), 156.19 (N–CO–O), 169.99 (O–CO–). IR (KBr) ν_{max} cm^{-1} 3410 s (OH); 3266 s (NH); 1727 vs (C=O) ester; 1714 vs (C=O) carbamate; 1555 s (amide II); 1456 s, 1395 s, 1367 s (CH_3); 1240 vs, 1159 vs (C-O-C); 3064 m, 3037 m, 1029 s, 700 s (ring). HRMS (ESI) calc for $C_{15}H_{21}O_5NNa$ $[M+Na]^+$ 318.13119, found: 318.13101.

tert-Butyl 2-(*S*)-(9-fluorenylmethyloxycarbonylamino)-3-hydroxypropanoate **5**

Z-L-Ser-O*t*Bu **4** (15.2 g; 51.5 mmol) in a glass pressure bottle was dissolved in 300 ml of methanol and 500 mg of 10 % Pd/C was added. The mixture was vigorously stirred and allowed to react under the atmosphere of hydrogen (15 psi) at rt overnight. TLC analysis revealed (toluene-ethyl acetate 50:50) that the starting compound had completely disappeared. The catalyst was filtered off through celite and the celite was washed with 300 ml of methanol. The filtrate was evaporated *in vacuo* to give 8.1 g of yellow residue, which was immediately dissolved in 150 ml of saturated solution of $NaHCO_3$. The flask was placed in the ice bath and Fmoc-OSu (17 g; 50.3 mmol) in 150 ml dioxane was added dropwise under stirring. After the addition of the total amount of acylation agent, stirring continued for 1 h at 0 °C and then overnight at room temperature. Thereafter, 250 ml of water was added and the reaction mixture was transferred to the separatory funnel and extracted 4 x with 150 ml of ethyl acetate. Combined organic layers were washed consecutively, once with 150 ml water, twice with 150 ml of brine and dried over anhydrous Na_2SO_4. Evaporation of the filtrate under reduced pressure furnished a colorless oil, which solidified upon standing at 5 °C. An analytical sample was prepared by crystallization from a mixture of diethyl ether-petroleum ether. Colorless solid. Yield 14.6 g (74% over two steps). m.p. 124-125 °C. R_f = 0.59 (toluene-ethyl acetate 50 : 50). $[\alpha]_D^{20}$ = +7.3 (c = 0.998; $CHCl_3$). ^{1}H NMR (600 MHz, DMSO): 1.39 (9H, s, $(CH_3)_3$), 4.01 (1H, ddd, J = 8.1, 5.9 and 4.5, >CH–N), 4.23 (1H, dd, J = 7.1 and 6.8, >CH–), 4.29 (1H, dd, J = 10.5

and 6.8, CO–O–C**<u>Ha</u>**Hb–), 4.33 (1H, dd, *J* = 10.5 and 7.1, CO–O–CHa**<u>Hb</u>**–), 4.65 (2H, m, –O–CH_2–), 4.88 (1H, t, *J* = 5.9, –OH), 7.43 (1H, d, *J* = 8.1, –NH–CO), 7.33 (2H, m, Ar-H), 7.42 (2H, m, Ar-H), 7.74 (2H, m, Ar-H), 7.89 (2H, m, Ar-H). ^{13}C NMR (150.9 MHz, DMSO): 27.87 ((CH_3)$_3$), 46.82 (>CH–), 57.48 (>CH–N); 61.57 (–CH_2–O), 65.88 (–CH_2–O–CO), 80.77 (O–**<u>C</u>**(CH_3)$_3$), 120.29(2), 125.45, 125.42, 127.25(2) and 127.82(2) (8x Ar =CH–), 140.92(2) 143.99 and 144.02 (4x Ar >C=), 156.24 (N–CO–O), 170.03 (O–CO–). IR (KBr) ν_{max} cm^{-1} 3403 m (OH); 1737 vs (C=O) ester; 1682 vs C=O (carbamate); 1540 vs (amide II); 1261 s, 1156 s (C-O-C); 3068 w, 3041 w, 1452 m, 1033 m, 764 s, 741 s (ring); 2978 m, 1395 m, 1371 m (CH_3). HRMS (ESI) calc for $C_{22}H_{26}O_5N$ [M+H]$^+$ 384.18055, found: 384.18058.

L-Val-O*t*Bu.TsOH **20**

L-Valine **19** (5.9 g; 50 mmol) was suspended in 70 ml of *tert*-butyl acetate, and 6.5 ml of 70 % $HClO_4$ was slowly added under ice-cooling. The ice bath was removed and the reaction mixture was allowed to react at rt overnight. The reaction mixture was poured into 200 ml of water; the separated aqueous layer was basified with K_2CO_3 until pH ~ 10 was reached and then extracted 3 x with 100 ml of diethyl ether. The combined organic phases were dried over Na_2SO_4, filtered and carefully evaporated to afford crude L-Val-O*t*Bu (7.6 g; 43.8 mmol). The slightly yellow oil was dissolved in 20 ml of methanol and the solution of TsOH.H_2O (8.4 g; 43.8 mmol) in 20 ml methanol was added in one portion. The solvent was evaporated and the product was crystallized from a mixture of i-PrOH-diethyl ether-hexane. Yield 9.3 g (63%). White solid, m. p. 138-140°C. $[\alpha]_D^{20}$ = +11.5 (c = 0.374 ; CH_3OH). ^{1}H NMR (600 MHz, DMSO): 0.95 (3H, d, *J* = 7.0, CH_3), 0.98 (3H, d, *J* = 7.0, CH_3), 1.46 (9H, s, (CH_3)$_3$), 2.12 (1H, m, >CH–), 2.29 (3H, br s, Ar–CH_3), 3.80 (1H, d, *J* = 4.2, >CH–N), 7.12 (2H, m, Ar-H), 7.48 (2H, m, Ar-H). ^{13}C NMR (150.9 MHz, DMSO): 17.60 (CH_3), 18.39 (CH_3), 21.00 (Ar–CH_3), 27.80 ((CH_3)$_3$), 29.60 (>CH–), 57.75 (>CH–N), 83.25 (O–**<u>C</u>**(CH_3)$_3$), 125.70(2) and 128.30(2) (4x Ar =CH–), 137.92 and 145.77 (2x Ar >C=), 168.20 (–O–CO–). IR (KBr) ν_{max} cm^{-1} 2971 m, 2934 m, 1474 m, 1369 m (CH_3); 1742 vs (C=O); 1254 s (C-O); 1618 m, 1521 m (NH_3^+); 3061 w, 3040 w, 1498 m, 1106 m, 1011 s, 849 m, 813 m, 676 s (ring); 1227 s, 1192 s, 1163 s, 1035 s (SO_3^-). HRMS (ESI) calc for $C_9H_{20}O_2N$ [M]$^+$ 174.14886, found: 174.14871.

(9-Fluorenylmethyloxycarbonyl)asparagine **25**

L-Asparagine **23** (5 g; 37.8 mmol, $[\alpha]_D^{20}$ = +27° – 31°, c = 13.2 in 1M HCl) was placed in a 1 L round-bottom flask, equipped with a magnetic spin bar, and dissolved in a solution of Na_2CO_3

(4 g; 37.8 mmol) in 50 ml of water. The flask was immersed in an ice cooling bath and Fmoc-OSu (12.8 g; 37.8 mol) in 70 ml of dioxane was added dropwise under vigorous stirring during a period of 30 min. When the addition of Fmoc-Osu was complete, the reaction mixture (a dense slurry) was allowed to react for 1 h at 0 °C and then overnight at room temperature. The reaction mixture was cooled again in an ice bath and concentrated HCl was added dropwise until pH ~ 0-1 was reached. The crystals were filtered off in a Büchner funnel and rinsed with 100 ml of chilled water. The filter cake was frozen and dried by lyophilization. The final purification was carried out by dissolving the solid in 100 ml of hot chloroform (60 °C). After cooling, the crystals were filtered off and washed with petroleum ether. Yield 11.6 g (87%). White solid, m.p. 181-183°C. R_f = 0.76 (ethyl acetate-acetone-methanol-water 4 : 1: 1: 1). $[\alpha]_D^{20}$ = -11.1 (c = 0.350 ; DMF). ^{1}H NMR (600 MHz, DMSO): 2.46 (1H, dd, *J* = 15.5 and 8.0, –C**Ha**Hb–CO), 2.56 (1H, dd, *J* = 15.5 and 5.2, –CHa**Hb**–CO), 4.22 (1H, m, >CH–), 4.27 (2H, m, –CH$_2$–O), 6.92 and 7.35 (2H, 2x br s, –CO–NH$_2$), 7.53 (1H, d, *J* = 8.4, –NH–CO), 7.33 (2H, m, Ar-H), 7.42 (2H, m, Ar-H), 7.71 (2H, m, Ar-H), 7.89 (2H, m, Ar-H), 12.59 (1H, br s, COOH). ^{13}C NMR (150.9 MHz, DMSO): 36.91 (–CH$_2$–), 46.78 (>CH–), 50.74 (>CH–N), 65.87 (–CH$_2$–O–CO), 120.29(2), 125.43, 125.45, 127.28(2) and 127.82(2) (8x Ar =CH–), 140.88(2), 143.97 and 144.00 (4x Ar >C=), 155.98 (N–CO–O), 171.36 (N–CO–), 173.36 (–COOH). IR (KBr) ν_{max} cm^{-1} 3405 s (NH); 3432 s, 3324 s (NH$_2$); 1735 vs (C=O) acid; 1697 vs (C=O) carbamate; 1657 vs (C=O) amide; 1541 s (amide II); 3065 m, 1478 m, 1211 s, 758 m, 739 s (ring). HRMS (ESI) calc for $C_{19}H_{18}O_5N_2Na[M+Na]^+$ 377.11079, found: 377.11084.

(9-Fluorenylmethyloxycarbonyl)glutamine **26**

Compound **18** was prepared by the reaction of L-Gln **24** (5 g; 34.2 mmol, $[\alpha]_D^{20}$ = +33°, c = 5 % in 5M HCl), and Fmoc-OSu (11.5 g; 34.2 mmol) by the method previously used for **25**. Yield 11.7 g (93%). R_f = 0.76 (ethyl acetate-acetone-methanol-water 4 : 1: 1: 1). $[\alpha]_D^{20}$ = -13.6 (c = 0.286 ; DMF). ^{1}H NMR (500 MHz, DMSO): 1.76 and 1.97 (2H, 2x m, –CH$_2$–), 2.16 (2H, t, *J* = 7.7, –CH$_2$–CO), 3.94 (1H, ddd, *J* = 9.5, 7.9 and 4.7, >CH–N), 4.21 (1H, m, >CH–), 4.26 (2H, m, –CH$_2$–O), 6.79 and 7.33 (2H, 2x br s, –CO–NH$_2$), 7.68 (1H, d, *J* = 7.9, –NH–CO), 7.33 (2H, m, Ar-H), 7.41 (2H, m, Ar-H), 7.72 (2H, m, Ar-H), 7.88 (2H, m, Ar-H). ^{13}C NMR (125.7 MHz, DMSO): 26.82 (–CH$_2$–), 31.68 (–CH$_2$–), 46.91 (>CH–), 53.77 (>CH–N), 65.98 (–CH$_2$–O–CO), 120.42(2), 125.60(2), 127.42(2) and 127.98(2) (8x Ar =CH–), 141.00(2) and 144.09(2) (4x Ar >C=), 156.48 (N–CO–O), 173.90 (–CO–NH$_2$), 174.07 (–COOH). IR (KBr)

ν_{max} cm^{-1} 3429 m, 3335 w (NH); 1724 vs (C=O) acid; 1698 vs (C=O) carbamate; 1658 vs (C=O) amide; 1532 m (amide II); 3066 w, 3040 w, 3021 w, 1670 w, 1450 m, 1215 m, 751 m, 740 m (ring). HRMS (ESI) calc for $C_{20}H_{20}O_5N_2Na[M+Na]^+$ 391.12644, found: 377.12656.

(*tert*-Butoxycarbonyl)asparagine **30**

Boc_2O (25.7 g; 0.1176 mol) in 150 ml of dioxane was added dropwise to an ice-cooled solution of L-asparagine **23** (14.9 g; 0.112 mol) and Na_2CO_3 (11.9 g; 0.112 mol) in 150 ml of water. The reaction mixture was stirred for 1 h at 0 °C and then at rt overnight. The volume of the reaction mixture was reduced to half; the slurry was cooled in an ice bath and pH was adjusted by the addition of an aqueous solution of conc. $KHSO_4$ to 2-3. The crystals were filtered off in a Büchner funnel and rinsed with 100 ml of chilled water. The filter cake was lyophilized and the pure product was gained by crystallization from methanol-ethyl acetate-petroleum ether mixture.

Intermediate **30** (18.9 g; 81.4 mmol) was suspended in a mixture of 90 ml of acetonitrile, 90 ml of ethyl acetate and 45 ml of water. Thereafter, (diacetoxyiodo)benzene (31.4 g; 97.7 mmol) was added in 5 portions within 15 minutes. Ten minutes after the addition of the total amount of (diacetoxyiodo) benzene, the slurry turned clear, followed by rapid precipitation of the crude product. The filter cake was washed out with 200 ml of chilled ethyl acetate and no additional purification was needed. Yield 12.7 g (77%). White solid, m.p. 209-211 °C. R_f = 0.40 (ethyl acetate-acetone-methanol-water 4 : 1: 1: 1). $[\alpha]_D^{20}$ = -5.3 (c = 0.318 ; acetic acid). ^{1}H NMR (600 MHz, DMSO): 1.39 (9H, s, $(CH_3)_3$), 2.77 (1H, dd, *J* = 11.9 and 9.1, –C**Ha**Hb–N), 3.01 (1H, dd, *J* = 11.9 and 5.4, –CHa**Hb**–N), 3.67 (1H, ddd, *J* = 9.1, 6.0 and 5.4, >CH–N), 6.28 (1H, br d, *J* = 6.0, –NH–CO). ^{13}C NMR (150.9 MHz, DMSO): 28.33 ($(CH_3)_3$), 40.65 ($–CH_2–N$), 51.08 (>CH–N), 78.42 (O–**C**$(CH_3)_3$), 155.35 (N–CO–O), 171.18 (–COOH). IR (KBr) ν_{max} cm^{-1} 3348 m (NH); 1714 vs (C=O) acid; 1684 vs (C=O) carbamate; 1530 m (amide II); 1624 s (NH_2); 2978 m, 2932 m, 1366 m (CH_3). HRMS (ESI) calc for $C_{18}H_{15}O_4N_2$ $[M-H]^+$ 203.10373, found: 203.10366.

Ac-β-azido-Ala-Val-Phe-$CONH_2$ **43**

Yield 115 mg (69%). Lyophilizate. $[\alpha]_D^{20}$ = -19.3 (c = 0.218; DMSO). ^{1}H NMR (600 MHz, DMSO): 0.74 (3H, d, *J* = 6.8, CH_3), 0.75 (3H, d, *J* = 6.8, CH_3), 1.87 (3H, s, CH_3–CO), 1.92 (1H, m, >CH–), 2.78 (1H, dd, *J* = 13.8 and 9.1, –C**Ha**Hb–), 2.99 (1H, dd, *J* = 13.8 and 5.2, –CHa**Hb**–), 3.38 (1H, dd, *J* = 12.6 and 8.0, –C**Ha**Hb–N_3), 3.46 (1H, dd, *J* = 12.6 and 4.7,

–CHa**Hb**–N_3), 4.09 (1H, dd, *J* = 8.6 and 6.6, >CH–N), 4.47 (1H, ddd, *J* = 9.1, 8.3 and 5.2, >CH–N), 4.58 (1H, td, *J* = 8.2, 8.0 and 4.7, >CH–N), 7.06 and 7.32 (2H, 2x br d, *J* = 2.0, $CONH_2$), 7.17 (1H, m, Ar-H), 7.22 (2H, m, Ar-H), 7.24 (2H, m, Ar-H), 7.90 (1H, d, *J* = 8.3, –NH–CO), 7.93 (1H, d, *J* = 8.6, –NH–CO), 8.31 (1H, d, *J* = 8.2, –NH–CO). ^{13}C NMR (150.9 MHz, DMSO): 18.10 (CH_3), 19.30 (CH_3), 22.67 (**C**H_3–CO), 30.64 (>CH–), 37.82 (–CH_2–), 51.75 (–CH_2–N_3), 52.38 (>CH–N), 53.70 (>CH–N), 58.15 (>CH–N), 126.42, 128.23(2) and 129.31(2) (5x Ar =CH–), 137.95 (Ar >C=), 169.22 (N–CO–), 169.86 (N–CO–), 170.44 (N–CO–), 172.89 (–$CONH_2$). IR (KBr) ν_{max} cm^{-1} 3423 s, 3292 s (NH); 2108 m (N_3); 1666 vs, 1642 vs (C=O); 1534 m (amide II); 3066 w, 3032 w, 1455 w, 1154 w, 700 w (ring); 2966 w, 2875 w (CH_3). HRMS (ESI) calc for $C_{19}H_{27}O_4N_7Na$ $[M+Na]^+$ 440.20167, found: 440.20177.

Ac-β-azido-Ala-Phe-Phe-$CONH_2$ **44**

Yield 141 mg (76%). Lyophilizate. $[\alpha]_D^{20}$ = -9.7 (c = 0.185; DMSO). ^{1}H NMR (600 MHz, DMSO): 1.83 (3H, s, CH_3–CO), 2.74 (1H, dd, *J* = 14.0 and 9.2, –C**Ha**Hb–), 2.95 (1H, dd, *J* = 14.0 and 4.7, –CHa**Hb**–), 2.82 (1H, dd, *J* = 13.8 and 8.7, –C**Ha**Hb–), 3.01 (1H, dd, *J* = 13.8 and 5.2, –CHa**Hb**–), 3.31 (1H, dd, *J* = 12.6 and 8.1, –C**Ha**Hb–N_3), 3.42 (1H, dd, *J* = 12.6 and 4.6, –CHa**Hb**–N_3), 4.43 (1H, ddd, *J* = 8.7, 8.3 and 5.2, >CH–N), 4.44 (1H, ddd, *J* = 9.2, 8.1 and 4.7, >CH–N), 4.48 (1H, ddd, *J* = 8.4, 8.1 and 4.6, >CH–N), 7.10 and 7.28 (2H, 2x br d, *J* = 2.0, $CONH_2$), 7.16 – 7.27 (10H, m, 2x C_6H_5), 8.02 (1H, d, *J* = 8.3, –NH–CO), 8.15 (1H, d, *J* = 8.1, –NH–CO), 8.20 (1H, d, *J* = 8.4, –NH–CO).^{13}C NMR (150.9 MHz, DMSO): 22.68 (**C**H_3–CO), 37.46 (–CH_2–), 37.80 (–CH_2–), 51.70 (–CH_2–N_3), 52.23 (>CH–N), 53.88 (>CH–N), 54.31 (>CH–N), 126.48, 126.50, 128.25(2), 128.28(2) and 129.40(4) (10x Ar =CH–), 137.68 and 137.92 (2x Ar >C=), 169.07 (N–CO–), 169.79 (N–CO–), 170.52 (N–CO–), 172.77 (–$CONH_2$). IR (KBr) ν_{max} cm^{-1} 3406 s, 3287 s (NH); 2107 m (N_3); 1666 vs, 1643 vs (C=O); 1538 m (amide II); 3064 w, 3031 w, 1498 w, 1454 w, 746 w, 700 w (ring). HRMS (ESI) calc for $C_{23}H_{26}O_4N_7$ $[M+1]^+$ 464.20518, found: 464.20460.

Ac-γ-azido-Dab-Phe-Phe-$CONH_2$ **45**

Yield 153 mg (80%). Lyophilizate. $[\alpha]_D^{20}$ = -25.4 (c = 0.358; DMSO). ^{1}H NMR (500 MHz, DMSO): 1.81 (3H, s, CH_3–CO), 1.63 and 1.75 (2H, 2x m, –CH_2–),2.75 (1H, dd, *J* = 13.9 and 9.4, –C**Ha**Hb–), 2.95 (1H, dd, *J* = 13.9 and 4.6, –CHa**Hb**–), 2.82 (1H, dd, *J* = 13.9 and 8.8, –C**Ha**Hb–), 3.01 (1H, dd, *J* = 13.9 and 5.1, –CHa**Hb**–), 3.20 (2H, m, –CH_2–N_3), 4.24 (1H,

ddd, *J* = 8.8, 7.8 and 5.1, >CH–N), 4.42 (1H, ddd, *J* = 8.8, 8.1 and 5.1, >CH–N), 4.44 (1H, ddd, *J* = 9.4, 8.0 and 4.6, >CH–N), 7.09 and 7.26 (2H, 2x d, *J* = 1.9, $CONH_2$), 7.16 – 7.27 (10H, m, 2x C_6H_5), 7.98 (1H, d, *J* = 8.0, –NH–CO), 8.02 (1H, d, *J* = 8.1, –NH–CO), 8.03 (1H, d, *J* = 7.8, –NH–CO). ^{13}C NMR (125.7 MHz, DMSO): 22.67 (**C**H_3–CO), 31.30 (–CH_2–), 37.38 (–CH_2–), 37.75 (–CH_2–), 47.64 (–CH_2–N_3), 50.31 (>CH–N), 53.96 (>CH–N), 54.09 (>CH–N), 126.45(2), 128.21(2), 128.27(2), 129.36(2) and 129.39(2) (10x Ar =CH–), 137.81 and 137.98 (2x Ar >C=), 169.68 (N–CO–), 170.75 (N–CO–), 171.07 (N–CO–), 172.80 (–$CONH_2$). IR (KBr) ν_{max} cm^{-1} 3419 s, 3293 s (NH); 2104 m (N_3); 1665 s, 1643 vs (C=O); 1530 m (amide II); 3064 w, 3031 w, 1498 w, 1455 w, 746 w, 701 w (ring); 2930 w, (CH_2). HRMS (ESI) calc for $C_{24}H_{29}O_4N_7Na$ $[M+Na]^+$ 502.21732, found: 502.21746.

Ac-δ-azido-Orn-Phe-Phe-$CONH_2$ **46**

Yield 152 mg (77%). Lyophilizate. $[\alpha]_D^{20}$ = -23.6 (c = 0.233; DMSO). ^1H NMR (600 MHz, DMSO): 1.81 (3H, s, CH_3–CO), 1.41 (2H, m, –CH_2–), 1.44 and 1.55 (2H, 2x m, –CH_2–), 2.75 (1H, dd, *J* = 14.0 and 9.4, –C**Ha**Hb–), 2.96 (1H, dd, *J* = 14.0 and 4.6, –CHa**Hb**–), 2.83 (1H, dd, *J* = 13.8 and 8.7, –C**Ha**Hb–), 3.00 (1H, dd, *J* = 13.8 and 5.2, –CHa**Hb**–), 3.24 (2H, m, –CH_2–N_3), 4.19 (1H, td, *J* = 7.8, 7.8 and 5.5, >CH–N), 4.42 (1H, ddd, *J* = 8.7, 8.3 and 5.2, >CH–N), 4.45 (1H, ddd, *J* = 9.4, 8.1 and 4.6, >CH–N), 7.09 and 7.24 (2H, 2x d, *J* = 1.9, $CONH_2$), 7.16 – 7.26 (10H, m, 2x C_6H_5), 7.977 (1H, d, *J* = 7.8, –NH–CO), 7.982 (1H, d, *J* = 8.1, –NH–CO), 7.989 (1H, d, *J* = 8.3, –NH–CO). ^{13}C NMR (150.9 MHz, DMSO): 22.67 (**C**H_3–CO), 24.88 (–CH_2–), 29.30 (–CH_2–), 37.38 (–CH_2–), 37.74 (–CH_2–), 50.60 (–CH_2–N_3), 52.26 (>CH–N), 53.97 (>CH–N), 54.08 (>CH–N), 126.44, 126.45, 128.21(2), 128.27(2) and 129.37(4) (10x Ar =CH–), 137.86 and 137.97 (2x Ar >C=), 169.62 (N–CO–), 170.78 (N–CO–), 171.53 (N–CO–), 172.80 (–$CONH_2$). IR (KBr) ν_{max} cm^{-1} 3397 m, 3283 m (NH); 2102 m (N_3); 1662 s, 1642 vs (C=O); 1543 m (amide II); 3065 w, 3031 w, 1498 w, 1455 w, 745 w, 700 w (ring); 2929 w, 2860 w (CH_2). HRMS (ESI) calc for $C_{25}H_{31}O_4N_7Na$ $[M+Na]^+$ 516.23297, found: 516.23296.

Ac-ω-azido-Lys-Phe-Phe-$CONH_2$ **47**

Yield 145 mg (71%). Lyophilizate. $[\alpha]_D^{20}$ = -24.5 (c = 0.326; DMSO). ^1H NMR (600 MHz, DMSO): 1.20 (2H, m, –CH_2–), 1.40 and 1.50 (2H, 2x m, –CH_2–), 1.45 (2H, m, –CH_2–), 1.81 (3H, s, CH_3–CO), 2.75 (1H, dd, *J* = 14.0 and 9.5, –C**Ha**Hb–), 2.95 (1H, dd, *J* = 14.0 and 4.7,

–CHa**<u>Hb</u>**–), 2.83 (1H, dd, *J* = 13.8 and 8.6, –C**<u>Ha</u>**Hb–), 3.00 (1H, dd, *J* = 13.8 and 5.1, –CHa**<u>Hb</u>**–), 3.24 (2H, m, $–CH_2–N_3$), 4.16 (1H, ddd, *J* = 8.6, 8.0 and 5.3, >CH–N), 4.42 (1H, ddd, *J* = 8.6, 8.0 and 5.1, >CH–N), 4.44 (1H, ddd, *J* = 9.5, 8.1 and 4.7, >CH–N), 7.10 and 7.24 (2H, 2x d, *J* = 2.0, $CONH_2$), 7.16 – 7.26 (10H, m, 2x C_6H_5), 7.94 (2H, 2x d, *J* = 8.0, 2x –NH–CO), 7.96 (1H, d, *J* = 8.1, –NH–CO). ^{13}C NMR (150.9 MHz, DMSO): 22.65 ($–CH_2–$), 22.66 (**<u>C</u>**H_3–CO), 28.11 ($–CH_2–$), 31.54 ($–CH_2–$), 37.34 ($–CH_2–$), 37.75 ($–CH_2–$), 50.66 ($–CH_2–N_3$), 52.56 (>CH–N), 53.92 (>CH–N), 54.07 (>CH–N), 126.40, 126.43, 128.19(2), 128.25(2), 129.34(2) and 129.36(2) (10x Ar =CH–), 137.88 and 137.98 (2x Ar >C=), 169.55 (N–CO–), 170.77 (N–CO–), 171.81 (N–CO–), 172.74 ($–CONH_2$). IR (KBr) ν_{max} cm^{-1} 3291 m (NH); 2099 m (N_3); 1665 s, 1640 vs (C=O); 1539 m (amide II); 3065 w, 3031 w, 1498 w, 1455 w, 745 w, 701 w (ring); 2934 w, 2861 w (CH_2). HRMS (ESI) calc for $C_{26}H_{33}O_4N_7Na$ $[M+Na]^+$ 530,24862 found: 530.24861.
